# Adverse Events Associated with Immune Checkpoint Blockade in Patients with Cancer: A Systematic Review of Case Reports

**DOI:** 10.1371/journal.pone.0160221

**Published:** 2016-07-29

**Authors:** Noha Abdel-Wahab, Mohsin Shah, Maria E. Suarez-Almazor

**Affiliations:** 1 Section of Rheumatology and Clinical Immunology, Department of General Internal Medicine, University of Texas MD Anderson Cancer Center, Houston, Texas, United States of America; 2 Rheumatology and Rehabilitation Department, Assiut University Hospitals, Assiut, Egypt; Exploratory Oncology Research & Clinical Trial Center, National Cancer Center, JAPAN

## Abstract

**Background:**

Three checkpoint inhibitor drugs have been approved by the US Food and Drug Administration for use in specific types of cancers. While the results are promising, severe immunotherapy-related adverse events (irAEs) have been reported.

**Objectives:**

To conduct a systematic review of case reports describing the occurrence of irAEs in patients with cancer following checkpoint blockade therapy, primarily to identify potentially unrecognized or unusual clinical findings and toxicity.

**Data Sources:**

We searched Medline, EMBASE, Web of Science, PubMed ePubs, and Cochrane CENTRAL with no restriction through August 2015.

**Study Selection:**

Studies reporting cases of cancer develop irAEs following treatment with anti CTLA-4 (ipilimumab) or anti PD-1 (nivolumab or pembrolizumab) antibodies were included.

**Data Extraction:**

We extracted data on patient characteristics, irAEs characteristics, how irAEs were managed, and their outcomes.

**Data Synthesis:**

191 publications met inclusion criteria, reporting on 251 cases. Most patients had metastatic melanoma (95.6%), and the majority were treated with ipilimumab (93.2%). Autoimmune colitis, hepatitis, endocrinopathies, and cutaneous irAEs were the most frequently reported irAEs in ipilimumab treated patients. A broad spectrum of toxicities were reported for almost every body system. Moreover, well-defined diseases such as sarcoidosis, polyarthritis, polymyalgia rheumatica/arteritis, lupus, celiac disease, dermatomyositis, and Vogt-Koyanagi-like syndrome were reported. The most frequent irAEs reported with anti-PD1 agents were dermatitis for pembrolizumab, and thyroid disease and pneumonitis for nivolumab. Complete resolution of adverse events occurred in most cases. However, persistent irAEs and death were reported, mainly in patients treated with ipilimumab.

**Limitations:**

Our study is limited by information available in the original reports.

**Conclusions:**

Evidence from case reports shows that cancer patients develop irAEs following checkpoint blockade therapy, and can occasionally develop clearly defined autoimmune systemic diseases. While discontinuation of therapy and/or treatment can result in resolution of irAEs, long-term sequelae and death have been reported.

## 1. Introduction

Advances in checkpoint blockade therapy have expanded our understanding of the complex interactions between the immune system, cancer cells, and their environment. T-cell activation is a key event in adaptive immunity, which when aberrant, can result in autoimmunity [1]. Most human cancers have evidence of adaptive immune dysregulation with genetic and epigenetic alterations in tumor cells, resulting in diverse antigenic expression that can elicit an immune activation. This immune activation is primarily T-cell mediated and regulated by stimulatory, co-stimulatory, and inhibitory (checkpoint) signals [2–4].

Immune-modulatory therapy can enhance antitumor immunity through various approaches. Currently, the most salient modality is the use of targeted monoclonal antibodies (mAb) against regulatory immune checkpoint molecules that inhibit T cell activation [5]. Three immune checkpoint inhibitors have been approved by the Food and Drug Administration (FDA) since 2011. Ipilimumab, was the first agent approved for advanced melanoma. It is a fully human IgG1 mAb that blocks the cytotoxic T lymphocyte antigen 4 (CTLA-4), a checkpoint inhibitor of T cell activation [6]. Pembrolizumab is the second immune checkpoint inhibitor therapy to receive FDA approval in 2014 for patients with advanced melanoma. It is an engineered humanized IgG4 mAb that regulates T cell activation by blocking programmed cell death protein 1 (PD-1). Nivolumab is another fully human IgG4 anti PD-1 mAb to receive approval by FDA for patients with advanced melanoma in 2014, and for patients with metastatic non-small cell lung cancer in 2015 [7]. The most recent FDA approval was received in September, 2015 for the combination of ipilimumab and nivolumab in patients with advanced melanoma. In addition, the efficacy of the checkpoint blockade with other agents or radiotherapy is currently being evaluated for various cancers [8].

Despite the impressive benefits of the immune checkpoint blockade, its use can be hampered by the occurrence of serious adverse events which can affect multiple organs of the body including skin, the gastrointestinal tract, the kidneys, both peripheral and central nervous systems, liver, lymph nodes, eyes, pancreas, and the endocrine system [9–13]. These immunotherapy-related adverse events (irAEs) can present in a wide variety of forms ranging from mild to severe, and can be fatal [14]. Many patients with irAEs require permanent discontinuation of treatment, and potentially long courses of corticosteroids, and even anti-tumor necrosis factor therapy to mitigate effects [15].

The most common irAEs in data pooled from clinical trials include hypophysitis, colitis, hepatitis, pneumonitis, and rash [16]. However, there are increasing case reports of patients who develop irAEs resembling inflammatory and rheumatic diseases such as arthritis, nephritis, myositis and polymyalgia-like syndromes [17–22]. Given the myriad of events that can occur with immune checkpoint blockade, we conducted a systematic review of case reports of irAEs, primarily to identify potentially unrecognized or unusual clinical findings and toxicity.

## 2. Materials and Methods

### 2.1 Data sources and searches

We searched five databases: Medline, EMBASE, Web of Science, PubMed ePubs, and The Cochrane Library–CENTRAL, with no language restrictions, up to August 2015. Manual bibliography search of the selected articles was also performed. Broad based search terms were used such as cancer, adverse or harmful events, checkpoint, and immune therapy to name a few. Search strategy is provided in S1 Appendix.

### 2.2 Study selection

Screening of eligible publications was carried out independently by two reviewers (NA and MS). First, by screening titles and abstracts, then by reviewing the full text of likely relevant articles. Disagreements were resolved by consensus. Original case reports reporting irAEs in patients with cancer following treatment with anti CTLA-4 or anti PD-1 antibodies were included if they provided a separate clinical description for each case reported. The FDA approved drugs ipilimumab, pembrolizuab, and nivolumab were considered whether the patients received the treatment in a clinical trial or as part of standard of care. We excluded case reports on non FDA approved checkpoint blockade therapy.

### 2.3 Data extraction

Data was extracted independently by two reviewers (NA and MS). For articles published in languages other than English, data was extracted using Google translator. We extracted data on patient characteristics (demographics, type of cancer, type of immune checkpoint blockade, number of doses), irAEs characteristics, and when reported, grade of toxicity according to the Common Terminology Criteria for Adverse Events (CTCAE) defined by the National Cancer Institute, management of irAEs, and outcomes.

### 2.4 Quality assessment

We used the guidelines for publishing adverse events reports recommended by the International Society for Pharmacoepidemiology (ISPE) and the International Society of Pharmacovigilance (ISoP) to evaluate the quality of the case reports [23]. The assessment was carried out independently by two investigators (NA and MS) addressing the items reported by the guidelines as required information, without regard to the items reported as desirable or relevant. The items appraised included: i) relevance of the title to the reported information, ii) adequate description of the patient (demographics, existing health condition, relevant past medical history, physical and laboratory abnormalities, and significant morbidity or mortality), iii) adequate description of the drug (identification of both the generic and trade names of the drug and the manufacturer, drug dosage, duration between drug administration and the reported adverse events, and concomitant therapy that could potentially contributes to the development of adverse events), iv) adequate description of the adverse events and their outcome, and v) discussion of the evidence supporting the causal association between the drug and the reported adverse events. Possible item ratings are yes, partially, or no; disagreement was resolved by consensus.

### 2.5 Data synthesis and analysis

Descriptive statistics were used to summarize data, with median and interquartile range for continuous variables and frequencies and percentages for dichotomous variables. The data was analyzed using Statistical Package for Social Sciences (SPSS) v 21.0 for Windows (SPSS, Inc., Chicago, IL, USA) [24].

## 3. Results

### 3.1 Publication characteristics

**Fig 1:**
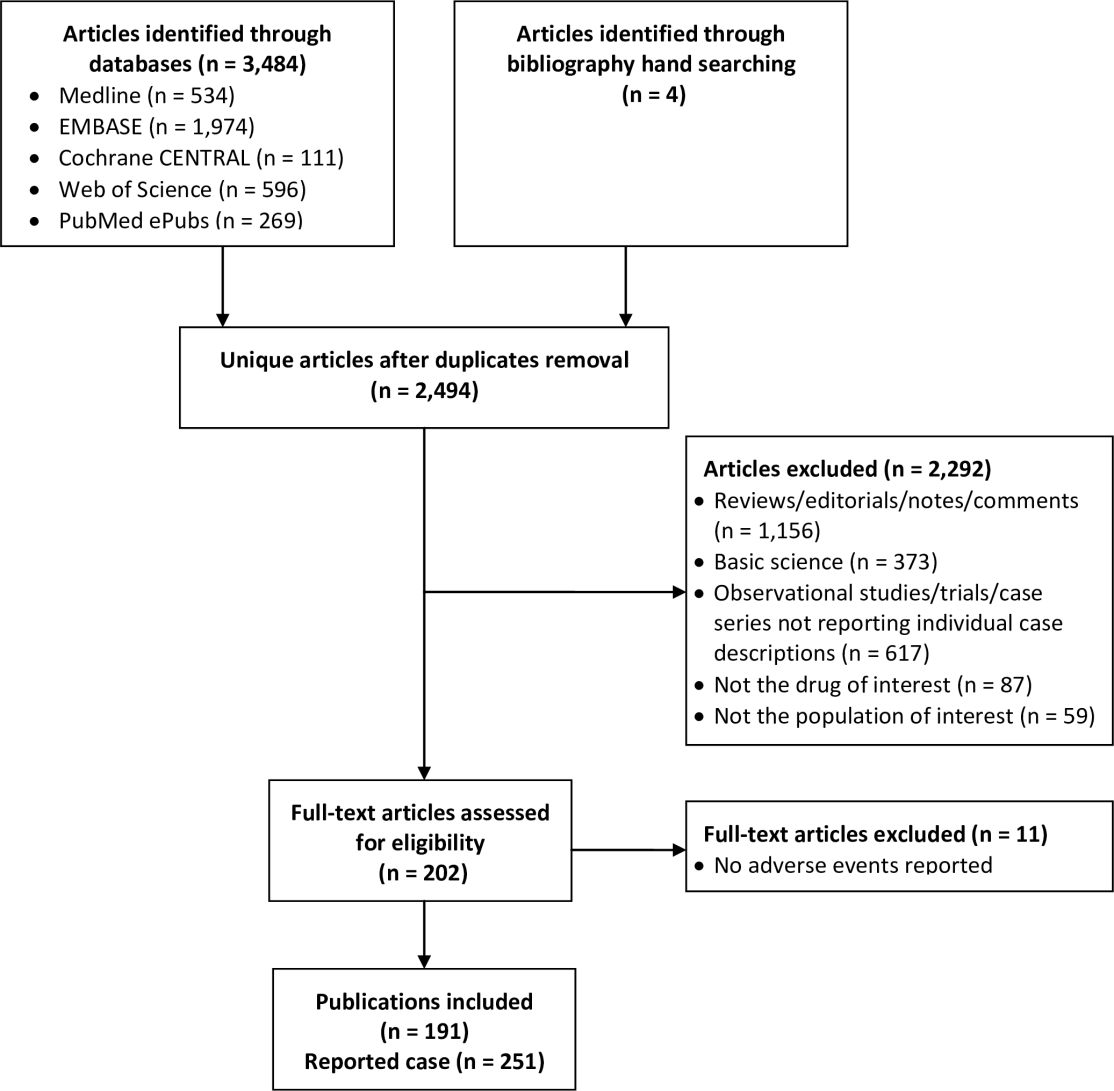
Study selection flowchart.

A total of 2,494 unique citations were initially retrieved, Fig 1. We identified, 202 citations as potentially relevant and reviewed the full publication. We excluded 11 publications reporting cases in which no adverse events occurred “S2 Appendix”. Thus, we included 191 publications (reporting on 251 cases with clinical description of each reported case provided separately). Cases from the United States were most common (53.4%), followed by Germany (8.4%), and France (6.4%).

### 3.2 Quality appraisal

The overall quality of the included cases was moderate to high “S3 Appendix”. Most cases had titles relevant to the reported information (97.6%), clear description of the patients’ demographics (95.6%), health status (94.4%), past medical history (93.6%), physical and laboratory investigations (93.6%), and significant morbidity or mortality (95.2%). The causative drug was clearly identified for most (96.8%), but the drug dosage was not provided in approximately one third of the cases. The duration between drug administration and the occurrence of the adverse events was generally specified (87.6%), as the potential influence of any concomitant therapy (94.4%). Accurate description of the severity of the events and their outcome was reported in 88.0%, and an appropriate discussion supporting a causal relationship between the drug and the adverse events was provided in 90.4%.

### 3.3 Patient characteristics (Table 1).

**Table 1:** Characteristics of the reported cases.

Patient characteristics	N (%)
**Median age**	60 (26–88) years^a^
**Male/Female**	154 (63.1)/90 (36.9)^a^
**Type of cancer**	
Metastatic melanoma	240 (95.6)
Prostate cancer	7 (2.8)
Lung cancer	3 (1.2)
Bladder cancer	1 (0.4)
**Type of checkpoint therapy**	
Ipilimumab	234 (93.2)^b^
Pembrolizumab	10 (4.0)
Nivolumab	7 (2.8)
**Time to first reported immunotherapy-related adverse events**‡	**n = 219**^**c**^
First dose	37 (16.9)
Second dose	61 (27.9)
Third dose	55 (25.1)
Fourth dose	54 (24.7)
Others	12 (5.4)

^a^The age was not reported in five cases, and the gender was not reported in seven cases.

^b^Total number included one patient who had sequential ipilimumab followed by pembrolizumab, and one patient who had concurrent ipilimumab and nivolumab.

^c^Number of doses before the first reported adverse events was not specified in 32 cases.

Median age of cases was 60 years (range 26–88 years), with male predominance (63.1%). Most patients had metastatic melanoma (95.6%). Other cancers sites included prostate, lung and bladder. Ipilimumab was the most frequently reported agent, in 234 cases, pembrolizumab in 10 and nivolumab in 7. Time to first reported adverse events after treatment was variable, most frequently after the second dose in 61 patients (27.9%), and the third and fourth doses in 55 (25.1%) and 54 patients (24.7%) respectively.

### 3.4 Immunotherapy-related adverse events (Table 2)

**Table 2:** Immunotherapy-related adverse events in the reported cases.

Immunotherapy-related adverse events	N (%)		
	Ipilimumab N = 234	Pembrolizumab N = 10	Nivolumab N = 7
**Gastrointestinal**	**93 (39.7)**^a^		
Colitis/enterocolitis	68 (29.1)		
Colitis complicated by intestinal perforation	12 (5.1)		
Hepatitis	17 (7.3)		
Pancreatitis	2 (0.9)		
**Endocrine**	**79 (33.7)**	**2 (20.0)**	**3 (42.9)**
Hypophysitis (manifested as panhypopitutarism)	68 (29.1)		
Thyrotoxicosis	4 (1.7)		1 (14.3)
Hypothyroid	4 (1.7)	1 (10.0)	2 (28.6)
Syndrome of inappropriate secretion of antidiuretic hormone	1 (0.4)		
Central adrenal insufficiency	1 (0.4)		
Primary adrenal insufficiency	1 (0.4)		
Diabetes Mellitus		1 (10.0)	
**Dermatologic**	**60 (25.6)**^b^	**5 (50.0)**	**2 (28.6)**
Rash	26 (11.1)		1 (14.3)
Pruritus	15 (6.4)		
Vitiligo	8 (3.4)	1 (10.0)	
Dermatitis	7 (3.0)	3 (30.0)^a^	
Sweet syndrome	3 (1.3)		
Drug eruption	2 (0.9)		
Poliosis	1 (0.4)		
Delayed hypersensitivity reaction	1 (0.4)		
Alopecia universalis	1 (0.4)		
Grover disease	1 (0.4)		
Pyoderma gangrenosum	1 (0.4)		
Toxic epidermal necrolysis	1 (0.4)		
Chronic non-caseation granuloma	1 (0.4)		
Bullous pemphigoid		1 (10.0)	
Psoriasis			1 (14.3)
**Ophthalmologic**	**24 (10.3)**^**c**^	**1 (10.0)**	
Uveitis	10 (4.3)	1 (10.0)	
Conjunctivitis	5 (2.1)		
Orbital inflammation	5 (2.1)		
Grave’s ophthalmology	2 (0.9)		
Choroidal neovascularization	2 (0.9)		
Optic neuropathy	2 (0.9)		
Keratitis	1 (0.4)		
Retinopathy	1 (0.4)		
**Neurologic**	**23 (9.8)**^**d**^	**1 (10.0)**	
Encephalopathy	4 (1.7)		
Guillian-Barre syndrome	3 (1.3)		
Polyradiculoneuropathy	2 (0.9)		
Symmetrical multifocal neuropathy	2 (0.9)		
Transverse myelitis	2 (0.9)		
Necrotizing myelopathy	2 (0.9)		
Myasthenia gravis	2 (0.9)		
Phrenic nerve palsy	2 (0.9)		
Immune related meningitis	1 (0.4)		
Meningioradiculoneuritis	1 (0.4)		
Peripheral neuropathy	1 (0.4)	1 (10.0)	
Autoimmune inner ear disease	1 (0.4)		
Multiple sclerosis	1 (0.4)		
Inflammatory enteric neuropathy	1 (0.4)		
**Hematologic**	**9 (3.8)**^**e**^		
Thrombocytopenia	3 (1.3)		
Pancytopenia	2 (0.9)		
Neutropenia	1 (0.4)		
Eosinophilia	1 (0.4)		
Pure red blood cell aplasia	1 (0.4)		
Acquired hemophilia A	1 (0.4)		
Disseminated intravascular coagulopathy	1 (0.4)		
**Genitourinary**	**9 (3.8)**		
Renal failure	5 (2.1)		
Acute/granulomatous interstitial nephritis	2 (0.9)		
Acute tubular necrosis	1 (0.4)		
Lymphocytic vasculitis of the uterus	1 (0.4)		
**Respiratory**	**6 (2.5)**	**1 (10.0)**	**3 (42.9)**^**f**^
Pneumonitis	5 (2.1)	1 (10.0)	3 (42.9)
Acute respiratory distress	1 (0.4)		2 (28.6)
**Musculoskeletal**	**4 (1.7)**^**g**^	**3 (30.0)**^**g**^	
Polyarthritis	1 (0.4)	2 (20.0)	
Arthralgia	1 (0.4)		
Myalgia	2 (0.9)	1 (10.0)	
Chronic granulomatous inflammation of rectus abdominis muscle	1 (0.4)		
Rhabdomyolysis		1 (10.0)	
**Cardiac**	**2 (0.9)**		
Pericarditis	1 (0.4)		
Takotsubo like syndrome	1 (0.4)		
**Defined systemic disease**	**16 (6.8)**	**1 (10.0)**	
Lung sarcoidosis	4 (1.7)		
Cutaneous and pulmonary sarcoidosis	3 (1.3)		
Polymyalgia rheumatica/ giant cell arteritis	2 (0.9)	1 (10.0)	
Muscular sarcoidosis	1 (0.4)		
Neurological and pulmonary sarcoidosis	1 (0.4)		
Celiac disease	1 (0.4)		
Lupus nephritis	1 (0.4)		
Dermatomyositis	1 (0.4)		
Autoimmune inflammatory myopathy	1 (0.4)		
Vogt-Koyanagi-like syndrome	1 (0.4)		

^a^Five patients developed colitis and hepatitis, and 1 patient developed colitis and pancreatitis, in the patient treated with sequential ipilimumab and pembrolizumab, colitis occurred before starting pembrolizumab and dermatitis after starting pembrolizumab.

^b^Two patients developed rash and pruritus, one patient developed rash and vitiligo, two patients developed pruritus and vitiligo, one patient developed rash, vitiligo, and pruritus, and one patient developed vitiligo and alopecia universalis.

^c^One patient developed keratitis with uveitis, one patient developed choroidal neovascularization with uveitis, and two patients developed optic neuropathy with uveitis.

^d^Two patients developed necrotizing myelopathy and encephalopathy, myasthenia gravis occurred in one patient after concurrent treatment with ipilimumab and nivolumab.

^e^Thrombocytopenia was complicated by disseminated intravascular coagulopathy in one patient.

^f^In two patients, pneumonitis was complicated by ARDS.

^g^One patient developed polyarthritis and myalgia both in ipilimumab and nivolumab groups.

#### Ipilimumab

In the 234 patients who had received ipilimumab, gastrointestinal irAEs were reported in 39.7% of the cases, primarily colitis (34.2%) of which 5.1% developed life threatening intestinal perforation. Autoimmune hepatitis was also reported in 17 patients (7.3%). Hypophysitis manifested as panhypopituitarism was the most commonly reported endocrine irAEs occurring in 29.1% of the cases, followed by thyrotoxicosis or hypothyroidism (4.0% each). Cutaneous irAEs were reported in 60 patients (25.6%), mainly rash and pruritus. Other less frequent irAEs including ophthalmologic, neurologic, hematologic, genitourinary, respiratory, musculoskeletal, and cardiac adverse events were also reported. In addition, well defined systemic autoimmune or inflammatory diseases were reported including sarcoidosis (of the lung, skin, nervous system or muscle), polymyalgia rheumatica/giant cell arteritis, celiac disease, lupus nephritis, dermatomyositis, autoimmune inflammatory myopathy, and Vogt-Koyanagi-like syndrome.

#### Pembrolizumab

Ten cases reported irAEs with pembrolizumab. In contrast to ipilimumab, gastrointestinal irAEs were not reported. Cutaneous irAEs were most common, primarily dermatitis, in 30.0% of the cases. Other manifestations included endocrine, ophthalmologic, neurologic, respiratory, and musculoskeletal adverse events. Polymyalgia rheumatica/giant cell arteritis was the only defined systemic autoimmune disease reported.

#### Nivolumab

Seven cases reported irAEs with nivolumab. Similar to pembrolizumab, gastrointestinal irAEs were not reported. Endocrine irAEs were reported in 3 patients (42.9%), primarily autoimmune thyroid disease. Pneumonitis was also reported in 42.9% of the cases, and was complicated by acute respiratory distress syndrome in 28.6%. Cutaneous irAEs were less frequent and defined systemic autoimmune diseases were not reported (Table 2).

The grade of toxicity was seldom reported (Table 3). Grade 3 toxicity was most frequently reported, primarily in patients who received ipilimumab and had gastrointestinal irAEs.

**Table 3:** Grade of toxicity in the reported cases.

Immunotherapy-related adverse events	N (%)		
	Ipilimumab N = 234	Pembrolizumab N = 10	Nivolumab N = 7
**Gastrointestinal (reported data)**	**n = 29**		
Grade 1	3 (10.3)		
Grade 2	5 (17.2)		
Grade 3	16 (55.2)		
Grade 4	5 (17.2)		
**Dermatologic (reported data)**	**n = 13**		
Grade 1	6 (46.2)		
Grade 2	5 (38.5)		
Grade 3	2 (15.3)		
**Endocrine (reported data)**	**n = 5**		
Grade 3	5 (100.0)		
**Respiratory (reported data)**	**n = 1**		**n = 3**
Grade 2			1 (33.3)
Grade 3	1 (100.0)		2 (66.7)
**Hematologic (reported data)**	**n = 3**		
Grade 3	1 (33.3)		
Grade 4	2 (66.7)		
**Ophthalmologic (reported data)**	**n = 2**		
Grade 1	2 (100.0)		
**Musculoskeletal (reported data)**	**n = 1**	**n = 1**	
Grade 2		1 (100.0)	
Grade 3	1 (100.0)		
**Neurologic (reported data)**	**n = 1**		
Grade 3	1 (100.0)		

### 3.5 Management and clinical outcomes (Table 4)

**Table 4:** Management and outcome of the reported adverse events.

Immunotherapy-related adverse events	N (%)		
	Ipilimumab N = 234	Pembrolizumab N = 10	Nivolumab N = 7
**Management (reported data)**	**n = 216**	**n = 9**	**n = 6**
No treatment required	8 (3.7)	1 (11.1)	
**Required treatment**	**208 (96.3)**	**8 (88.9)**	**6 (100.0)**
Corticosteroids	189 (90.9)	4 (50.0)	4 (66.7)
Replacement therapy	52 (25.0)	2 (25.0)	2 (33.3)
Infliximab	19 (9.1)		2 (33.3)
DMARDs (Tacrolimus, HCQ, MMF, SSZ, CYC A)^a^	11 (5.3)	2 (25.0)	
**Outcome of the adverse events (reported data)**	**n = 214**^**b**^	**n = 7**	**n = 6**
Resolution of adverse events	151 (70.6)	4 (57.1)	5 (83.3)
Persistent sequelae	52 (24.3)	3 (42.9)	
Death secondary to adverse events	10 (4.7)		1 (16.7)
**Discontinuation of checkpoint therapy because of adverse events (reported data)**	**n = 111**	**n = 6**	**n = 5**
Yes (permanent or temporary)	63 (56.8)	3 (50.0)	2 (40.0)
No	41 (36.9)	3 (50.0)	2 (40.0)
Course completed before adverse events	7 (6.3)		1 (20.0)

^a^HCQ: hydroxychloroquine; MMF: mycophenolate mofetil; SSZ: sulfasalazine; CYC A: cyclosporine A.

^b^One patient died before further investigation due to underlying cardiomyopathy with fatal arrhythmia.

#### Ipilimumab

Details of treatment were available for 216 cases. In 8 cases (3.7%), no treatment was required and spontaneous resolution of the irAEs was observed. In contrast, 208 patients (96.3%) required treatment: 189 patients (90.9%) received corticosteroids, 19 infliximab (9.1%), and 11 disease modifying anti-rheumatic drugs (DMARDs) or immunomodulatory agents (5.3%). Hormonal replacement therapy was required for 52 (25.0%) patients.

Data on irAEs outcomes was available for 214 cases. Resolution of the adverse events was reported in 151 cases (70.6%), persistent symptoms were reported in 52 cases (24.3%), and death as a result of complicated irAEs was reported in 10 (4.7%). Sixty-three patients (56.8%), discontinued ipilimumab, permanently or temporarily. Treatment rechallenge was reported in one case who develop severe thrombocytopenia complicated by disseminated intravascular coagulopathy [25]. Supportive treatment was given until the symptoms resolved, and the patient was able to continue additional doses.

#### Pembrolizumab

Treatment was reported in 9 cases with 8 requiring treatment. Resolution of the adverse events was reported in 4 cases (57.1%), and persistent symptoms in 3 (42.9%). Half of the cases required discontinuation of therapy. Treatment rechallenge was reported in one case who developed polyarthritis [17]. Salazopyrin, opioid, and pamidronate were given concurrently upon rechallange, with no exacerbation of polyarthritis.

#### Nivolumab

Treatment was reported for 6 patients, all of them requiring treatment. Resolution of the adverse events was reported in 5 cases (83.3%), and death secondary to the irAEs was reported in one. Two cases required discontinuation of therapy. Treatment rechallenge was reported in one case who developed pneumonitis [26]. Oral steroids were given until the symptoms resolved, and the patient was able to complete his treatment period for two-years.

## 4. Discussion

Immune checkpoint blockade therapy is increasingly being used in patients with cancer, primarily melanoma, and is being evaluated for a number of other cancers. However, irAEs can limit their use, and can result in serious adverse outcomes including death. While some adverse events have been well described in clinical trials (e.g. dermatitis, colitis), increasingly, other inflammatory and autoimmune manifestations are being reported. Case reports can provide vital clues and signals in identifying rare but serious events, and can generate hypotheses that can direct ongoing scientific research. We conducted a systematic review of case reports of patients treated with checkpoint blockade to identify the scope of manifestations that may occur with these therapies, especially rare events, only including publications that had adequate description of the clinical manifestations of the patients reported.

Our review primarily found reports of patients treated with ipilimumab, as would be expected, since this therapy was approved earlier than anti-PD1 agents. Many of the publications reported well-recognized irAEs that were identified earlier in clinical trials, such as dermatitis, and endocrine manifestations for all therapies, and gastrointestinal events, primarily enterocolitis, for ipilimumab. Although less frequently, many other manifestations not clearly identified previously in clinical trials were reported, including for instance, potentially inflammatory or potentially autoimmune complications such as various types of neuropathy or myelitis in the nervous system, ophthalmologic manifestations such as uveitis or retinopathy, among others. Overall, potential toxicity was reported for almost every body system or organ including cutaneous, ophthalmologic, neurologic, hematologic, genitourinary, respiratory, musculoskeletal, and cardiac adverse events. Moreover, several publications reported sporadic *de novo* occurrence of various syndromes compatible with well-defined diseases such as sarcoidosis, lupus, psoriasis, diabetes, and polymyalgia rheumatic/arteritis among others.

There were fewer cases reported for pembrolizumab and nivolumab compared to ipilimumab. Cutaneous irAEs were the most frequently reported events in pembrolizumab treated patients, and autoimmune thyroid disease and pneumonitis in patients treated with nivolumab. Enterocolitis was not reported with either agent. While this lower frequency of published case reports can be attributed in part to the later approval of anti-PD1 agents compared to ipilimumab, there is some evidence that these agents may have fewer adverse events [27–31].

The severity of the irAEs was not clearly specified in the majority of the reported cases, but many patients had grade 3 toxicity. Longer-term sequelae or death were occurred in close to one third of patients, predominantly in patients treated with ipilimumab who developed colitis with intestinal perforation. The most common treatment for irAEs was corticosteroids, but infliximab and other immunomodulatory drugs were occasionally administered, and many patients had to discontinue therapy. The suggested treatment algorithm for irAEs has been recently discussed in two reviews [32–33] highlighting the role of corticosteroids as a cornerstone for treatment, and recommending more aggressive immunosuppressant in refractory cases. From their experience, Dadu et al. recommended physiologic steroid replacement, instead of high dose corticosteroids, for the majority of cases with hypohysitis and no signs of persistent inflammation, and early use of infliximab in colitis to shorten the duration of treatment with corticosteroids and improve clinical outcome [32]. Michot et al. raised the unresolved issue of a possible detrimental effect of corticosteroids and other immunosuppressant drugs on the efficacy of checkpoint blockade on tumor progression [33]. In both reviews, early consultation of appropriate specialists, and discontinuation of the checkpoint blockade with grade 3–4 toxicity were recommended [32, 33]. While it is conceivably that discontinuation could result in worse cancer outcomes, it is also possible that patients suffering irAEs may have enhanced beneficial immune anti-tumoral responses compared to those who do not develop toxicity [34].

To our knowledge, this is the largest and most comprehensive systematic review of case reports of irAEs related to CTLA-4 and PD-1 blockade therapy. Previously, Betrand et al. published a systematic review and meta-analysis of irAEs with CTLA-4 blockade, including both ipilimumab and tremelimumab (non-approved) [9]. They pooled data from clinical trials including 1265 patients, and described 100 case reports separately. However, their search strategy was more limited and only included cases up to February 2014. All of their included cases with adequate clinical descriptions are included in our review. The most common irAEs identified in their meta-analysis of trials involved the skin and gastrointestinal tract. Ibraham et al. [35] reviewed the results of 14 trials of ipilimumab including 1498 cancer patients, and reported similar results.

Checkpoint blockade immunotherapy has revolutionized the treatment of cancer, with impressive survival benefits attained through up regulation of the antitumoral immune response. Nevertheless, blocking of regulatory checkpoint molecules can also result in aberrant immune activation leading to undesirable off-target inflammation and autoimmunity [15]. Once a T cell is activated, CTLA-4 (CD28 homolog) is expressed on its surface to block the costimulatory signal through its greater affinity for CD80/86, playing an important role in self-tolerance and therefore limiting autoimmunity under normal homeostatic conditions. Competitive inhibition of CTLA-4 by ipilimumab results in persistent T cell activation, increased cytokine production, and enhanced T cell mediated immune responses [36, 37]. Likewise, the PD-1 receptor is expressed on the surface of activated T cells, B cells, T regulatory, and natural killer cells. It delivers inhibitory signals that limit the response of activated T cells by binding to programmed cell death ligands 1 and 2 (PD-L1 and PD-L2) on the surface of activated immune cells and non-hematopoietic stem cells. PD-L1 and PD-L2 are expressed in various tumor cells, which could partly explain the ability of tumor cells to evade immune surveillance. Therefore, blocking this pathway can enhance antitumor immune responses [7]. Cumulative evidence from clinical trials, case reports, and case series demonstrate that cancer patients treated with these agents develop a wide myriad of inflammatory and irAEs in various organs, ranging from mild to life-threatening [11–15]. It is thought that most irAEs, especially the severe ones, arise from nonspecific immune activation, however, for some effects the underlying etiology is poorly understood (e.g. eosinophilia). Why different patients may develop toxicity in different organs is unclear. While many genetic determinants have been described in patients with primary autoimmune diseases, it is uncertain whether patients developing irAEs share the same genetic risks [9]. Furthermore, it is also unclear whether treatment with these agents may result in the unveiling of underlying ‘silent’ autoimmunity resulting in chronic, persistent inflammatory disease. In addition, other personal ecological factors such as the microbiome may also play a role in determining susceptibility to specific irAEs, such as enterocolitis [38]. The recognition of potential risk factors would assist in identifying patients who may be poor candidates for this novel therapeutic modality, or may require additional surveillance and prompt aggressive treatment when developing an adverse event.

Our systematic review is based on a comprehensive literature search of five databases with no restrictions, with defined criteria for inclusion and quality assessment. However, it is limited by the quality of data available in the reports. We included case reports in abstracts even when a full publication was not available. Because case reports are not research studies, there are no systematic ways of searching for unpublished relevant cases. Case reports of adverse events are likely to report unique, unusual, or severe features, and therefore may not be representative of the population of interest at large and cannot be used to infer overall frequency or severity. However, they can provide important safety signals, and a reference for clinicians and researchers so they can seek and identify previously unrecognized adverse events.

## 5. Conclusion

In spite of its considerable benefit in patients with cancer, immune checkpoint blockade can be limited by the occurrence of irAEs that can be life threatening. The broad spectrum of irAEs reported warrant the need for controlled longitudinal studies to confirm the association of therapy with rare, severe events, and to identify individual and genetic determinants of potential autoimmunity, which could result in severe toxicity.

## Supporting Information

S1 Appendix MEDLINE Search Strategy. (PDF)

S2 Appendix Full-text excluded articles and reasons for exclusion. (PDF)

S3 Appendix Reported cases and their quality appraisal. (PDF)

S4 Appendix PRISMA Checklist. (PDF)
