# Supplementary material for: Adverse Events Associated with Immune Checkpoint Blockade in Patients with Cancer: A Systematic Review of Case Reports
Source: PLoS One. 2016 Jul 29;11(7):e0160221. doi: 10.1371/journal.pone.0160221 (PMC4966895; doi:10.1371/journal.pone.0160221)
Supplement: S2 Appendix — (PDF) [file pone.0160221.s002.pdf]

S2 Appendix. Full-text excluded articles and reasons for exclusion

**Authors**	**Year**	**Reasons for exclusion**
Du Four (1)	2014	No immunotherapy-related adverse events were reported
Gomez-Valent (2)	2012	No immunotherapy-related adverse events were reported
Hodi (3)	2008	Insufficient information to determine if case had an immunotherapy-related adverse events
Idris (4)	2013	No immunotherapy-related adverse events were reported
Kyi (5)	2014	No immunotherapy-related adverse events were reported<br>Patients had pre-existing autoimmune diseases (multiple sclerosis, and rheumatoid arthritis)
Minter (6)	2013	No immunotherapy-related adverse events were reported<br>Patient had pre-existing Hepatitis C virus infection with elevated transaminases
Ranganath (7)	2015	No immunotherapy-related adverse events were reported
Ravi (8)	2014	No immunotherapy-related adverse events were reported, or insufficient information to determine if case had an immunotherapy-related adverse events<br>Patient had pre-existing Hepatitis C or B virus infections with elevated transaminases
Schindler (9, 10)*	2012	No immunotherapy-related adverse events were reported
Sharma (11)	2013	No immunotherapy-related adverse events were reported<br>Patient had pre-existing Hepatitis C virus infection with elevated transaminases

*Two publications for the same case reports.

References

1. Du Four S, Hong A, Chan M, Charakidis M, Duerinck J, Wilgenhof S, et al. Symptomatic histologically proven necrosis of brain following stereotactic radiation and ipilimumab in six lesions in four melanoma patients. Case Reports in Oncological Medicine. 2014;2014(417913).
2. Gomez-Valent M, Frias Lopez MC, Pontes Garcia C, Montero Leno AB, Cabre Serres M, Raich Montiu L, et al. A fatal event in a patient receiving Ipilimumab for metastasic melanoma: Causality analyses. European Journal of Hospital Pharmacy: Science and Practice. 2012;19 (2):179.
3. Hodi FS, Oble DA, Drappatz J, Velazquez EF, Ramaiya N, Ramakrishna N, et al. CTLA-4 blockade with ipilimumab induces significant clinical benefit in a female with melanoma metastases to the CNS. Nature clinical practice Oncology. 2008;5(9):557-61.
4. Idris AF, Martin MJ, Davidson IR, O'Sullivan GJ, Jamaludin AA, Donnellan PP. Ocular melanoma liver metastases treated by percutaneous hepatic perfusion with melphalan followed by ipilimumab: A case report. Journal of Clinical Oncology. 2013;1).
5. Kyi C, Carvajal RD, Wolchok JD, Postow MA. Ipilimumab in patients with melanoma and autoimmune disease. Journal for Immunotherapy of Cancer. 2014;2(1):35.
6. Minter S, Willner I, Shirai K. Ipilimumab-induced hepatitis C viral suppression. Journal of Clinical Oncology. 2013;31(19):e307-e8.
7. Ranganath HA, Panella TJ. Administration of Ipilimumab to a Liver Transplant Recipient With Unresectable Metastatic Melanoma. Journal of Immunotherapy. 2015;38(5):211-.
8. Ravi S, Spencer K, Ruisi M, Ibrahim N, Luke JJ, Thompson JA, et al. Ipilimumab administration for advanced melanoma in patients with pre-existing Hepatitis B or C infection: a multicenter, retrospective case series. Journal for Immunotherapy of Cancer. 2014;2(1):33.
9. Schindler K, Kunstfeld R, Pehamberger H, Harmankaya K. An exceedingly rare case of primary rhabdoid melanoma of the urinary bladder. JDDG - Journal of the German Society of Dermatology. 2011;9 (9):774.
10. Schindler K, Schicher N, Kunstfeld R, Pehamberger H, Toepker M, Haitel A, et al. A rare case of primary rhabdoid melanoma of the urinary bladder treated with ipilimumab, an anti-CTLA 4 monoclonal antibody. Melanoma Research. 2012;22(4):320-5.
11. Sharma A, Thompson JA, Repaka A, Mehnert JM. Ipilimumab Administration in Patients With Advanced Melanoma and Hepatitis B and C. Journal of Clinical Oncology. 2013;31(21):E370-E2.
