# Supplementary material for: Adverse Events Associated with Immune Checkpoint Blockade in Patients with Cancer: A Systematic Review of Case Reports
Source: PLoS One. 2016 Jul 29;11(7):e0160221. doi: 10.1371/journal.pone.0160221 (PMC4966895; doi:10.1371/journal.pone.0160221)
Supplement: S3 Appendix — (PDF) [file pone.0160221.s003.pdf]

S3 Appendix. Reported cases and their quality appraisal

Authors	Year	Title	Patient demographics	Current health status	Medical history	Physical exam	Patient disposition	Drug identification	Dosage	Drug reaction interface	Concomitant therapy	Adverse events	Discussion
Abdallah (1)	2015	Yes	Yes	Yes	Yes	Yes	Yes	Yes	Yes	Yes	Yes	Yes	Yes
Ahmad (2)	2012	Partial	Yes	Yes	Yes	Yes	Yes	Yes	Yes	Yes	Yes	Yes	Yes
Ahmed (3)	2013	Yes	Yes	Partial	Partial	Partial	Yes	Yes	No	Yes	Partial	Yes	Partial
		Yes	Yes	Partial	Partial	Partial	Yes	Yes	No	Yes	Partial	Yes	Partial
		Yes	Yes	Partial	Partial	Partial	Yes	Yes	No	Yes	Partial	Partial	Partial
Akhtari (4)	2009	Yes	Yes	Yes	Yes	Yes	Yes	Yes	Yes	Yes	Yes	Yes	Yes
Anderson (5)	2013	Partial	Yes	Yes	Yes	Yes	Yes	Yes	Partial	Yes	Yes	Partial	Yes
Ashworth (6)	2014	Partial	Yes	Yes	Yes	Yes	Yes	Yes	Yes	No	Yes	Partial	Partial
Assi (7)	2013	Partial	Yes	Yes	Yes	Yes	Yes	Yes	Partial	Partial	Yes	Yes	Yes
		Partial	Yes	Yes	Yes	Yes	Yes	Yes	Partial	Yes	Yes	Yes	Yes
Audemard (8)	2013	Yes	Yes	Yes	Yes	Yes	Yes	Partial	No	No	Yes	Partial	Partial
Babi (9)	2013	Yes	Yes	Yes	Yes	Yes	Yes	Yes	Partial	Yes	Yes	Yes	Yes
Balakan (10)	2012	Yes	Yes	Yes	Yes	Yes	Yes	Yes	Yes	Yes	Yes	Yes	Yes
Barjaktarevic (11, 12)*	2013	Yes	Yes	Yes	Yes	Yes	Yes	Yes	No	Partial	Yes	Yes	Yes
Barnard (13)	2012	Yes	Yes	Yes	Yes	Yes	Yes	Yes	Yes	Yes	Yes	Yes	Yes
Bellutti (14)	2013	Yes	Partial	Partial	Partial	Partial	Partial	Partial	Partial	Partial	Partial	Partial	Partial
Berthod (15)	2012	Yes	Yes	Yes	Yes	Yes	Yes	Yes	Yes	Yes	Yes	Yes	Yes
Bhatia (16)	2009	Yes	Yes	Yes	Yes	Yes	Yes	Yes	Yes	Yes	Yes	Yes	Yes
Bompaire (17)	2012	Yes	Yes	Yes	Yes	Yes	Yes	Yes	Yes	Yes	Yes	Yes	Yes
Borodic (18-20)*	2014	Yes	Yes	Yes	Yes	Yes	Yes	Yes	Partial	Yes	Yes	Yes	Yes
Bot (21)	2013	Yes	Yes	Yes	Yes	Yes	Yes	Yes	Partial	Yes	Yes	Yes	Yes
		Yes	Yes	Yes	Yes	Yes	Yes	Yes	Partial	Yes	Yes	Yes	Yes
		Yes	Yes	Yes	Yes	Yes	Yes	Yes	Partial	Yes	Yes	Yes	Yes
Bot (22)	2012	Yes	Yes	Yes	Yes	Yes	Yes	Yes	Yes	Yes	Yes	Yes	Yes
Boyer (23)	2015	Yes	Yes	Yes	Yes	Yes	Yes	Yes	Yes	Yes	Yes	Yes	Yes
Bryce (24)	2013	Yes	Yes	Yes	Yes	Yes	Yes	Yes	Yes	Yes	Yes	Yes	Yes
Burke (25)	2011	Yes	Yes	Yes	Yes	Yes	Yes	Yes	Yes	Yes	Yes	Yes	Yes
Carlos (26)	2015	Yes	Yes	Yes	Yes	Yes	Yes	Yes	Yes	Yes	Yes	Yes	Yes

Carpenter (27)	2009	Yes	Yes	Yes	Yes	Yes	Yes	Yes	Yes	Yes	Yes	Yes	Yes
		Yes	Yes	Yes	Yes	Yes	Yes	Yes	Yes	Yes	Yes	Yes	Yes
		Yes	Yes	Yes	Yes	Yes	Yes	Yes	Yes	Yes	Yes	Yes	Yes
Cavalcante (28)	2015	Yes	Yes	Yes	Yes	Yes	Yes	Yes	Yes	Yes	Yes	Yes	Yes
		Yes	Yes	Yes	Yes	Yes	Yes	Yes	Yes	Yes	Yes	Yes	Yes
Cecchini (29)	2015	Yes	Yes	Yes	Yes	Yes	Yes	Yes	Yes	Yes	Yes	Yes	Yes
Chan (30)	2015	Yes	Yes	Yes	Yes	Yes	Yes	Yes	Yes	Yes	Yes	Yes	Yes
		Yes	Yes	Yes	Yes	Yes	Yes	Yes	Yes	Yes	Yes	Yes	Yes
Chargari (31)	2013	Yes	Yes	Yes	Yes	Yes	Yes	Yes	Yes	Yes	Yes	Yes	Yes
Chmiel (32)	2011	Yes	Yes	Yes	Yes	Yes	Yes	Yes	Yes	Yes	Yes	Yes	Yes
Conry (33)	2015	Yes	Yes	Yes	Yes	Yes	Yes	Yes	Yes	Yes	Yes	Yes	Yes
Crews (34)	2015	Yes	Yes	Yes	Yes	Yes	Yes	Yes	Yes	Yes	Yes	Yes	Partial
Crosson (35)	2015	Yes	Yes	Yes	Yes	Yes	Yes	Yes	Yes	Yes	Yes	Yes	Yes
De Felice (36)	2013	Yes	Yes	Yes	Yes	Yes	Yes	Yes	No	Yes	Yes	Yes	Partial
		Yes	Yes	Yes	Yes	Yes	Yes	Yes	No	Yes	Yes	Yes	Partial
		Yes	Yes	Yes	Yes	Yes	Yes	Yes	No	Yes	Yes	Yes	Partial
De Holland (37)	2013	Yes	Partial	Partial	Partial	Partial	Partial	Yes	No	Yes	Partial	Partial	Yes
		Yes	Partial	Partial	Partial	Partial	Partial	Yes	No	Yes	Partial	Partial	Yes
		Yes	Partial	Partial	Partial	Partial	Partial	Yes	No	Yes	Partial	Partial	Yes
De Sousa (38)	2014	Yes	Partial	Partial	Partial	Partial	Partial	Partial	No	Yes	Yes	Yes	Partial
		Yes	Partial	Partial	Partial	Partial	Partial	Partial	No	Yes	Yes	Yes	Partial
Delyon (39)	2011	Yes	Yes	Yes	Yes	Yes	Yes	Yes	Yes	Yes	Yes	Yes	Partial
Di Giacomo (40)	2009	Yes	Yes	Yes	Yes	Yes	Yes	Yes	Yes	Yes	Yes	Yes	Yes
		Yes	Yes	Yes	Yes	Yes	Yes	Yes	Yes	Yes	Yes	Yes	Yes
Dick (41)	2015	Yes	Yes	Yes	Yes	Yes	Yes	Yes	Yes	Yes	Yes	Yes	Yes
		Yes	Yes	Yes	Yes	Yes	Yes	Yes	Yes	Yes	Yes	Yes	Yes
		Yes	Yes	Yes	Yes	Yes	Yes	Yes	Yes	Yes	Yes	Yes	Yes
Dillard (42)	2010	Yes	Yes	Yes	Yes	Yes	Yes	Yes	Yes	Yes	Yes	Yes	Yes
		Yes	Yes	Yes	Yes	Yes	Yes	Yes	Yes	Yes	Yes	Yes	Yes
Dilling (43)	2014	Yes	Yes	Yes	Yes	Yes	Yes	Yes	No	Yes	Yes	Yes	No
Du Four (44)	2012	Yes	Yes	Yes	Yes	Yes	Yes	Yes	Yes	Yes	Yes	Yes	Yes
Du Rusquec (45)	2014	Yes	Yes	Yes	Yes	Yes	Yes	Yes	No	Yes	Yes	Yes	Yes
Eckert (46)	2009	Yes	Yes	Partial	Partial	Partial	Partial	Yes	Partial	Partial	Partial	Yes	Partial
Eranki (47)	2012	Yes	Yes	Yes	Yes	Yes	Yes	Yes	Yes	Yes	Yes	Yes	Yes
Eryilmaz (48)	2015	Yes	Yes	Yes	Yes	Yes	Yes	Yes	Yes	Yes	Yes	Yes	Yes

Fadel (49)	2009	Yes	Yes	Yes	Yes	Yes	Yes	Yes	Yes	Yes	Yes	Yes	Yes
Fischli (50)	2014	Yes	Yes	Yes	Yes	Yes	Yes	Yes	Yes	Yes	Yes	Yes	Yes
Forde (51)	2012	Yes	Yes	Yes	Yes	Yes	Yes	Yes	Yes	Yes	Yes	Yes	Yes
Franzen (52)	2013	Yes	Yes	Yes	Yes	Yes	Yes	Yes	Partial	Yes	Yes	Yes	Partial
Gao (53)	2014	Yes	Yes	Yes	Partial	Partial	Yes	Yes	Yes	Yes	Yes	Yes	Yes
		Yes	Yes	Yes	Partial	Partial	Yes	Yes	Yes	Yes	Yes	Yes	Yes
Garcia-Varona (54)	2013	Yes	Yes	Yes	Yes	Yes	Yes	Yes	No	Yes	Yes	Yes	Yes
Gaudy-Marqueste (55)	2013	Yes	Yes	Yes	Yes	Yes	Yes	Yes	Yes	Yes	Yes	Yes	Yes
Geisler (56)	2015	Yes	Yes	Yes	Yes	Yes	Yes	Yes	Yes	Yes	Yes	Yes	Yes
Gentile (57)	2013	Yes	Yes	Yes	Yes	Yes	Yes	Yes	Yes	Yes	Yes	Yes	Yes
Gerdes (58)	2014	Yes	Yes	Yes	Yes	Yes	Yes	Yes	Yes	No	Yes	Yes	Yes
Gil (59)	2013	Partial	Yes	Yes	Yes	Yes	Yes	Yes	No	Yes	Yes	Yes	Partial
Gilardi (60)	2014	Yes	Yes	Yes	Yes	Yes	Partial	Yes	No	No	Partial	Partial	Partial
Goethals (61)	2011	Yes	No	Partial	Partial	Partial	Partial	Partial	Partial	Partial	Yes	Partial	No
Goldstein (62, 63)*	2014	Yes	Yes	Yes	Yes	Yes	Yes	Yes	Yes	Yes	Yes	Yes	Yes
		Yes	Yes	Yes	Yes	Yes	Yes	Yes	Yes	Yes	Yes	Yes	Yes
Gordon (64)	2009	Yes	Yes	Yes	Yes	Yes	Yes	Yes	Yes	Yes	Yes	Yes	Yes
Gorka (65)	2014	Yes	Yes	Yes	Yes	Yes	Yes	Yes	Yes	Yes	Yes	Yes	Yes
Gormley (66)	2014	Yes	Yes	Yes	Yes	Yes	Yes	No	Yes	Yes	Yes	Yes	Yes
Haider (67)	2014	Yes	Yes	Yes	Yes	Yes	Yes	Yes	No	Yes	Yes	Partial	Yes
Hanrahan (68)	2013	Yes	Yes	Yes	Yes	Yes	Yes	Yes	No	Yes	Yes	Partial	Yes
		Yes	Yes	Partial	Partial	Partial	Partial	Yes	No	Yes	Partial	Partial	Partial
Harmankaya (69)	2011	Yes	Yes	Yes	Yes	Yes	Yes	Yes	Yes	Yes	Yes	Yes	Yes
Henderson (70)	2014	Yes	Yes	Yes	Yes	Yes	Yes	Yes	Yes	Yes	Yes	Yes	Yes
Herlopian (71)	2015	Yes	Yes	Yes	Yes	Yes	Yes	Yes	No	Yes	Yes	Yes	Yes
		Yes	Yes	Yes	Yes	Yes	Yes	Yes	No	Yes	Yes	Yes	Yes
Hinds (72)	2014	Yes	Yes	Yes	Yes	Yes	Yes	Yes	No	Yes	Yes	Yes	Partial
Hrgovic (73)	2015	Yes	Yes	Yes	Yes	Yes	Yes	Yes	No	Yes	Yes	Yes	Yes
Hundorfean (74)	2012	Yes	Yes	Yes	Yes	Yes	Yes	Yes	No	Yes	Yes	Partial	Partial
Hunter (75)	2009	Yes	Yes	Yes	Yes	Yes	Yes	Yes	No	Yes	Yes	Yes	Yes
Izzedine (76)	2014	Yes	Yes	Yes	Yes	Yes	Yes	Yes	Yes	Yes	Yes	Yes	Yes
		Yes	Yes	Yes	Yes	Yes	Yes	Yes	Yes	Yes	Yes	Yes	Yes

Jinnur (77)	2015	Yes	Yes	Yes	Yes	Yes	Yes	Yes	No	Yes	Yes	Yes	Yes
Johnson (78)	2013	Yes	Yes	Yes	Yes	Yes	Yes	Yes	No	No	Yes	Partial	Yes
Joseph (79)	2015	Yes	Yes	Yes	Yes	Yes	Yes	Yes	Yes	Yes	Yes	Partial	Yes
		Yes	Yes	Yes	Yes	Yes	Yes	Yes	Yes	Yes	Yes	Yes	Yes
		Yes	Yes	Yes	Yes	Yes	Yes	Yes	Yes	Yes	Yes	Yes	Yes
Juszczak (80)	2012	Yes	Yes	Yes	Yes	Yes	Yes	Yes	No	Yes	Yes	Yes	Yes
Kaehler (81)	2009	Yes	Yes	Yes	Yes	Yes	Yes	Yes	Yes	Yes	Yes	Yes	Yes
Kamil (82)	2013	Yes	Yes	Yes	Yes	Yes	Yes	Yes	Yes	Yes	Yes	Yes	Yes
Khirfan (83)	2014	Yes	Yes	Yes	Yes	Yes	Yes	Yes	No	Yes	Yes	Yes	Yes
Kleiner (84)	2012	Yes	Yes	Yes	Yes	Yes	Yes	Yes	No	Yes	Yes	Yes	Yes
		Yes	Yes	Yes	Yes	Yes	Yes	Yes	No	Yes	Yes	Yes	Yes
		Yes	Yes	Yes	Yes	Yes	Yes	Yes	No	Yes	Yes	Yes	Yes
		Yes	Yes	Yes	Yes	Yes	Yes	Yes	No	Yes	Yes	Yes	Yes
		Yes	Yes	Yes	Yes	Yes	Yes	Yes	Yes	Yes	Yes	Yes	Yes
Koch (85)	2012	Yes	Yes	Partial	Partial	Partial	Partial	Yes	No	Yes	Yes	Partial	Partial
Kopecky (86)	2015	Yes	Yes	Yes	Yes	Yes	Yes	Yes	Yes	Yes	Yes	Yes	Yes
Kwun (87)	2012	Yes	Yes	Yes	Yes	Yes	Yes	Yes	No	Yes	Yes	Yes	Yes
Kyllo (88)	2014	Yes	Yes	Yes	Yes	Yes	Yes	Yes	Yes	Yes	Yes	Yes	Yes
Lambert (89)	2015	Yes	Yes	Yes	Yes	Yes	Yes	Yes	Yes	Yes	Yes	Yes	Yes
Lammert (90)	2013	Yes	Yes	Yes	Yes	Yes	Yes	Yes	Yes	Yes	Yes	Yes	Yes
		Yes	Yes	Yes	Yes	Yes	Yes	Yes	Yes	Yes	Yes	Yes	Yes
		Yes	Yes	Yes	Yes	Yes	Yes	Yes	Yes	Yes	Yes	Yes	Yes
		Yes	Yes	Yes	Yes	Yes	Yes	Yes	Yes	Yes	Yes	Yes	Yes
		Yes	Yes	Yes	Yes	Yes	Yes	Yes	Yes	Yes	Yes	Yes	Yes
		Yes	Yes	Yes	Yes	Yes	Yes	Yes	Yes	Yes	Yes	Yes	Yes
		Yes	Yes	Yes	Yes	Yes	Yes	Yes	Yes	Yes	Yes	Yes	Yes
Lecouflet (91, 92)*	2013	Yes	Yes	Yes	Yes	Yes	Yes	Yes	No	Yes	Yes	Yes	Yes
Ledezma (93)	2011	Yes	Partial	Yes	Yes	Yes	Yes	Yes	Yes	Yes	Yes	Yes	Yes
		Yes	Yes	Yes	Yes	Yes	Yes	Yes	Yes	Yes	Yes	Yes	Yes
		Yes	Yes	Yes	Yes	Yes	Yes	Yes	Yes	Yes	Yes	Yes	Yes
		Yes	Yes	Yes	Yes	Yes	Yes	Yes	Yes	Yes	Yes	Yes	Yes
Leonard (94)	2013	Yes	Yes	Yes	Yes	Yes	Yes	Yes	Yes	Yes	Yes	Yes	Yes
Liao (95, 96)*	2014	Yes	Yes	Yes	Yes	Yes	Yes	Yes	No	Yes	Yes	Yes	Yes
		Yes	Yes	Yes	Yes	Yes	Yes	Yes	No	Yes	Yes	Yes	Yes
		Yes	Yes	Yes	Yes	Yes	Yes	Yes	No	Yes	Yes	Yes	Yes

Lim (97)	2008	Yes	Yes	Yes	Yes	Yes	Yes	Yes	No	Yes	Yes	Yes	Yes
Lipson (98)	2014	Yes	Yes	Yes	Yes	Yes	Yes	Yes	No	No	Yes	Yes	Yes
Loochtan (99)	2015	Yes	Yes	Yes	Yes	Yes	Yes	Yes	No	No	Partial	Yes	Yes
Ludlow (100)	2015	Yes	Yes	Yes	Yes	Yes	Yes	Yes	No	Yes	Yes	Yes	Yes
Luke (101)	2015	Yes	Yes	Yes	Yes	Yes	Yes	Yes	Yes	Yes	Yes	Yes	Yes
Lyall (102)	2012	Yes	Yes	Yes	Yes	Yes	Yes	Yes	No	Yes	Yes	Yes	Yes
Mahzari (103)	2015	Yes	Yes	Yes	Yes	Yes	Yes	Yes	Yes	Yes	Yes	Yes	Yes
		Yes	Yes	Yes	Yes	Yes	Yes	Yes	Yes	Yes	Yes	Yes	Yes
		Yes	Yes	Yes	Yes	Yes	Yes	Yes	Yes	Yes	Yes	Yes	Yes
		Yes	Yes	Yes	Yes	Yes	Yes	Yes	Yes	Yes	Yes	Yes	Yes
		Yes	Yes	Yes	Yes	Yes	Yes	Yes	Yes	Yes	Yes	Yes	Yes
		Yes	Yes	Yes	Yes	Yes	Yes	Yes	Yes	Yes	Yes	Yes	Yes
Mailleux (104)	2015	Yes	Yes	Yes	Yes	Yes	Yes	Yes	No	Partial	Yes	Yes	Yes
Majchel (105)	2015	Yes	Yes	Yes	Yes	Yes	Yes	Yes	Yes	Yes	Yes	Partial	Yes
Manousakis (106)	2013	Yes	Yes	Yes	Yes	Yes	Yes	Yes	Yes	Yes	Yes	Yes	Yes
Manusow (107)	2015	Yes	Yes	Yes	Yes	Yes	Yes	Yes	Yes	Yes	Yes	Yes	Yes
Marlier (108)	2014	Yes	Yes	Yes	Yes	Yes	Yes	Yes	Yes	Yes	Yes	Yes	Yes
		Yes	Yes	Yes	Yes	Yes	Yes	Yes	Yes	Yes	Yes	Yes	Yes
		Yes	Yes	Yes	Yes	Yes	Yes	Yes	Yes	Yes	Yes	Yes	Yes
		Yes	Yes	Yes	Yes	Yes	Yes	Yes	Yes	Yes	Yes	Yes	Yes
Martinez-Balzano (109)	2012	Yes	Yes	Yes	Yes	Yes	Yes	Yes	No	Yes	Yes	Yes	Yes
Martin-Liberal (110)	2015	Yes	Yes	Yes	Yes	Yes	Yes	Yes	Yes	Yes	Yes	Yes	Yes
Maur (111)	2012	Yes	Yes	Yes	Yes	Yes	Yes	Yes	Yes	Yes	Yes	Yes	Yes
McElnea (112)	2014	Yes	Yes	Yes	Yes	Yes	Yes	Yes	Yes	Yes	Yes	Yes	Yes
Merrill (113)	2014	Yes	Yes	Yes	Yes	Yes	Yes	Yes	Yes	Yes	Yes	Yes	Yes
Min (114)	2014	Yes	Yes	Yes	Yes	Yes	Yes	Yes	Yes	Yes	Yes	Yes	Yes
Min (115)	2013	Yes	Yes	Yes	Yes	Yes	Yes	Yes	Yes	Yes	Yes	Yes	Yes
Min (116)	2011	Yes	Yes	Yes	Yes	Yes	Yes	Yes	Yes	Yes	Yes	Yes	Yes
Minor (117)	2015	Yes	Yes	Yes	Yes	Yes	Yes	No	No	No	No	Yes	Yes
		Yes	Yes	Yes	Yes	Yes	Yes	No	No	No	No	Yes	Yes
Minor (118)	2013	Yes	Yes	Yes	Yes	Yes	Yes	Yes	Yes	Yes	Yes	Yes	Yes
Minor (119)	2009	Yes	Yes	Yes	Yes	Yes	Yes	Yes	Yes	Yes	Yes	Yes	Yes

		Yes	Yes	Yes	Yes	Yes	Yes	Yes	Yes	Yes	Yes	Yes	Yes
		Yes	Yes	Yes	Yes	Yes	Yes	Yes	Yes	Yes	Yes	Yes	Yes
Mis (120)	2013	Yes	Yes	Yes	Yes	Yes	Yes	Yes	Yes	Yes	Yes	Yes	Yes
Miserocchi (121)	2015	Yes	Yes	Yes	Yes	Yes	Yes	Yes	Yes	Yes	Yes	Yes	Yes
Mitchell (122)	2013	Yes	Yes	Yes	Yes	Yes	Yes	Yes	No	Yes	Yes	Yes	Yes
		Yes	Yes	Yes	Yes	Yes	Yes	Yes	No	Yes	Yes	Partial	Yes
		Yes	Yes	Yes	Yes	Yes	Yes	Yes	No	Yes	Yes	Yes	Yes
Modjtahedi (123)	2013	Yes	Yes	Yes	Yes	Yes	Yes	Yes	Partial	Yes	Yes	Yes	Yes
Morales (124)	2015	Yes	Yes	Yes	Yes	Yes	Yes	Yes	Yes	Yes	Yes	Yes	Yes
Muhammad (125)	2012	Yes	Yes	Yes	Yes	Yes	Yes	Yes	No	Yes	Yes	Yes	Yes
Munoz (126)	2014	Yes	Yes	Yes	Yes	Yes	Yes	Yes	Yes	Yes	Yes	Yes	Yes
Murakami (127)	2013	Yes	Yes	Yes	Yes	Yes	Yes	Yes	No	Yes	Yes	Partial	Yes
Murer (128)	2013	Yes	Yes	Yes	Yes	Yes	Yes	Yes	Yes	Yes	Yes	Partial	Yes
Murphy (129)	2014	Yes	Yes	Yes	Yes	Yes	Yes	Yes	No	Yes	Yes	Yes	Yes
Nallapaneni (130)	2014	Yes	Yes	Yes	Yes	Yes	Yes	Yes	Yes	Yes	Yes	Yes	Yes
Nancey (131)	2012	Yes	Yes	Yes	Yes	Yes	Yes	Yes	Yes	Yes	Yes	Yes	Yes
Narita (132)	2015	Yes	Yes	Yes	Yes	Yes	Yes	Yes	Yes	Yes	Yes	Yes	Yes
		Yes	Yes	Yes	Yes	Yes	Yes	Yes	Yes	Yes	Yes	Yes	Yes
Nishino (133)	2015	Yes	Yes	Yes	Yes	Yes	Yes	Yes	Yes	Yes	Yes	Yes	Yes
		Yes	Yes	Yes	Yes	Yes	Yes	Yes	Yes	Yes	Yes	Yes	Yes
		Yes	Yes	Yes	Yes	Yes	Yes	Yes	Yes	Yes	Yes	Yes	Yes
Noirez (134)	2011	Yes	Yes	Yes	Yes	Yes	Yes	Yes	Yes	Yes	Yes	Yes	Yes
Ohtsuka (135)	2015	Yes	Yes	Yes	Yes	Yes	Yes	Yes	Yes	Yes	Yes	Yes	Yes
O'Kane (136)	2014	Yes	Yes	Yes	Yes	Yes	Yes	Yes	Yes	Yes	Yes	Yes	Yes
Pages (137)	2013	Yes	Partial	Yes	Yes	Yes	Yes	Yes	Yes	Yes	Yes	Partial	Yes
Pedersen (138)	2014	Yes	Yes	Yes	Yes	Yes	Yes	Yes	Yes	No	Yes	Partial	Yes
Penumetsa (139)	2013	Yes	Yes	Yes	Yes	Yes	Yes	Yes	Yes	Yes	Yes	Partial	Yes
Pintova (140)	2013	Yes	Yes	Yes	Yes	Yes	Yes	Yes	Yes	Yes	Yes	Yes	Yes

Plachouri (141, 142)*	2012	Yes	Yes	Yes	Yes	Yes	Yes	Yes	No	Yes	Yes	Yes	Yes
Pocha (143)	2014	Yes	Yes	Yes	Yes	Yes	Yes	Yes	No	Yes	Yes	Yes	Yes
Postow (144)	2012	Yes	Yes	Yes	Yes	Yes	Yes	Yes	Yes	No	Yes	Yes	Yes
Rastogi (145)	2015	Yes	Yes	Yes	Yes	Yes	Yes	Yes	No	Yes	Yes	Yes	Yes
		Yes	Yes	Yes	Yes	Yes	Yes	Yes	No	Yes	Yes	Yes	Yes
		Yes	Yes	Yes	Yes	Yes	Yes	Yes	No	Yes	Yes	Yes	Yes
		Yes	Yes	Yes	Yes	Yes	Yes	Yes	No	Yes	Yes	Yes	Yes
		Yes	Yes	Yes	Yes	Yes	Yes	Yes	No	Yes	Yes	Yes	Yes
		Yes	Yes	Yes	Yes	Yes	Yes	Yes	No	Yes	Yes	Yes	Yes
		Yes	Yes	Yes	Yes	Yes	Yes	Yes	No	Yes	Yes	Yes	Yes
Ratuapli (146)	2014	Yes	Yes	Yes	Yes	Yes	Yes	Yes	No	No	Yes	Yes	Yes
Reule (147)	2013	Yes	Yes	Yes	Yes	Yes	Yes	Yes	Yes	Yes	Yes	Partial	Yes
Robinson (148)	2004	Yes	Yes	Yes	Yes	Yes	Yes	Yes	Yes	Partial	Yes	Yes	Yes
		Yes	Yes	Yes	Yes	Yes	Yes	Yes	Yes	Partial	Yes	Yes	Yes
Rodrigues (149)	2014	Yes	Yes	Yes	Yes	Yes	Yes	Yes	Yes	Yes	Yes	Yes	Yes
		Yes	Yes	Yes	Yes	Yes	Yes	Yes	Yes	Yes	Yes	Yes	Yes
Rudolph (150, 151)*	2014	Yes	Yes	Yes	Yes	Yes	Yes	Yes	Yes	Yes	Yes	Yes	Yes
Saenger (152)	2008	Yes	Yes	Yes	Yes	Yes	Yes	Yes	No	No	Yes	Yes	Yes
		Yes	Yes	Yes	Yes	Yes	Yes	Yes	No	No	Yes	Yes	Yes
Sampath (153)	2013	Yes	Yes	Yes	Yes	Yes	Yes	Yes	No	No	Yes	Yes	Yes
Schartz (154)	2010	Yes	Yes	Yes	Yes	Yes	Yes	Yes	No	No	Yes	Yes	Yes
Schleder (155)	2013	Yes	Partial	Yes	Yes	Yes	Yes	Yes	Yes	Yes	Yes	Yes	Yes
Shah (156)	2014	Yes	Yes	Yes	Yes	Yes	Yes	Yes	No	Yes	Yes	Yes	Yes
Sheikh Ali (157)	2015	Yes	Yes	Yes	Yes	Yes	Yes	Yes	Yes	Yes	Yes	Yes	Yes
Shivaprasad (158)	2013	Yes	Partial	Yes	Yes	Yes	Yes	Yes	No	No	Yes	Yes	Yes
Slingerland (159)	2012	Yes	Yes	Yes	Yes	Yes	Yes	Yes	Yes	Yes	Yes	Yes	Yes
Sohrab (160)	2013	Yes	Yes	Yes	Yes	Yes	Yes	Yes	Yes	Yes	Yes	Yes	Yes
Sprung (161)	2014	Yes	Yes	Yes	Yes	Yes	Yes	Yes	No	Partial	Yes	Yes	Yes

Sun (162)	2008	Yes	Yes	Yes	Yes	Yes	Yes	Yes	Yes	Yes	Yes	Yes	Yes
Thaipisuttikul (163, 164)*	2015	Yes	Yes	Yes	Yes	Yes	Yes	Yes	No	Yes	Yes	Yes	Yes
		Yes	Yes	Yes	Yes	Yes	Yes	Yes	Partial	Yes	Yes	Yes	Yes
Thajudeen (165, 166)*	2015	Yes	Yes	Yes	Yes	Yes	Yes	Yes	Yes	Yes	Yes	Yes	Yes
Thomsen (167)	2012	Yes	Yes	Yes	Yes	Yes	Yes	Yes	No	No	Yes	Partial	Yes
		Yes	Yes	Yes	Yes	Yes	Yes	Yes	No	No	Yes	Partial	Yes
Tissot (168)	2013	Yes	Yes	Yes	Yes	Yes	Yes	Yes	Yes	Yes	Yes	Yes	Yes
Tiu (169)	2015	Yes	Yes	Yes	Yes	Yes	Yes	Yes	Yes	Yes	Yes	Yes	Yes
		Yes	Yes	Yes	Yes	Yes	Yes	Yes	Yes	Yes	Yes	Yes	Yes
Toumeh (170)	2015	Yes	Yes	Yes	Yes	Yes	Yes	Yes	Yes	Yes	Yes	Yes	Yes
Tsaknakis (171)	2014	Yes	Yes	Yes	Yes	Yes	Yes	Yes	Yes	Yes	Yes	Yes	Yes
Tsiaras (172)	2011	Yes	Yes	Yes	Yes	Yes	Yes	Yes	No	No	Yes	Yes	Yes
Uslu (173)	2015	Yes	Yes	Yes	Yes	Yes	Yes	Yes	Yes	Yes	Yes	Yes	Yes
Van der Hiel (174)	2013	Yes	Yes	Partial	Partial	Partial	Partial	Yes	Yes	Yes	Partial	Yes	Partial
Van Rooij (175)	2013	Yes	Yes	Yes	Yes	Yes	Yes	Yes	Yes	Yes	Yes	Yes	Yes
Venditti (176)	2015	Yes	Yes	Yes	Yes	Yes	Yes	Yes	Yes	Yes	Yes	Yes	Yes
Victoria Martinez (177)	2014	Yes	Yes	Yes	Yes	Yes	Yes	Yes	No	No	Yes	Partial	Yes
Vogel (178)	2012	Yes	Yes	Yes	Yes	Yes	Yes	Yes	Yes	Yes	Yes	Yes	Yes
Voskens (179)	2012	Yes	Yes	Yes	Yes	Yes	Yes	Yes	Yes	Yes	Yes	Yes	Yes
Wigenhof (180)	2012	Yes	Yes	Yes	Yes	Yes	Yes	Yes	Yes	Yes	Yes	Yes	Yes
		Yes	Yes	Yes	Yes	Yes	Yes	Yes	Yes	Yes	Yes	Yes	Yes
		Yes	Yes	Yes	Yes	Yes	Yes	Yes	Yes	Yes	Yes	Yes	Yes
Wilgenhof (181)	2012	Yes	Yes	Yes	Yes	Yes	Yes	Yes	Yes	Yes	Yes	Yes	Yes
Wilgenhof (182)	2011	Yes	Yes	Yes	Yes	Yes	Yes	Yes	Yes	Yes	Yes	Yes	Yes
Wilgenhof (183)	2011	Yes	Yes	Yes	Yes	Yes	Yes	Yes	Yes	Yes	Yes	Yes	Yes
Wozniak (184)	2015	Yes	Yes	Yes	Yes	Yes	Yes	Yes	Yes	Yes	Yes	Yes	Yes

Wyluda (185)	2015	Yes	Yes	Yes	Yes	Yes	Yes	Yes	No	No	Yes	Partial	Yes
Yarze (186)	2013	Yes	Yes	Yes	Yes	Yes	Yes	Yes	Yes	Yes	Yes	Partial	Yes
Yeh (187)	2015	Yes	Yes	Yes	Yes	Yes	Yes	Yes	Yes	Yes	Yes	Yes	Yes
Yu (188)	2015	Yes	Yes	Yes	Yes	Yes	Yes	Yes	No	Yes	Yes	Yes	Yes
Yuan (189)	2010	Yes	Yes	Yes	Yes	Yes	Yes	Yes	Yes	No	Yes	Yes	Yes
Yun (190)	2015	Yes	Yes	Yes	Yes	Yes	Yes	Yes	Yes	Yes	Yes	Yes	Yes
Zmeili (191)	2013	Yes	Yes	Yes	Yes	Yes	Yes	Yes	Partial	Yes	Yes	Yes	Yes

*Two publications for the same case reports.

References

1. Abdallah AO, Herlopian A, Ravilla R, Bansal M, Chandra-Reddy S, Mahmoud F, et al. Ipilimumab-induced necrotic myelopathy in a patient with metastatic melanoma: A case report and review of literature. Journal of oncology pharmacy practice : official publication of the International Society of Oncology Pharmacy Practitioners. 2015.
2. Ahmad S, Lewis M, Corrie P, Iddawela M. Ipilimumab-induced thrombocytopenia in a patient with metastatic melanoma. J Oncol Pharm Pract. 2012;18(2):287-92.
3. Ahmed MK, Rein V, Shenker Y, Albertini M, Davis DB. Central adrenal insufficiency due to ipilimumab (Yervoy). Endocrine Reviews. 2013;1).
4. Akhtari M, Waller EK, Jaye DL, Lawson DH, Ibrahim R, Papadopoulos NE, et al. Neutropenia in a patient treated with ipilimumab (anti-CTLA-4 antibody). Journal of Immunotherapy. 2009;32(3):322-4.
5. Anderson L, Bhatia V. Ipilimumab immune-related adverse reactions: a case report. S D Med. 2013;66(8):315-7.
6. Ashworth MT, Daud A. Management of a patient with advanced BRAF-mutant melanoma. JNCCN Journal of the National Comprehensive Cancer Network. 2014;12(3):315-9.
7. Assi H, Wilson KS. Immune toxicities and long remission duration after ipilimumab therapy for metastatic melanoma: two illustrative cases. Curr. 2013;20(2):e165-9.
8. Audemard A, de Raucourt S, Miocque S, Comoz F, Giraud JM, Dreno B, et al. Melanoma-associated retinopathy treated with ipilimumab therapy. Dermatology. 2013;227(2):146-9.
9. Babi C, Jaclyn S, Mark N, John H. Ipilimumab induced enterocolitis: A fatal immune-related adverse event of melanoma treatment. Journal of Gastroenterology and Hepatology Research. 2013;2(12):934-6.
10. Balakan O, Suner A, Yigiter R, Balakan T, Sirikci A, Sevinc A. Long-term survival in metastatic malignant melanoma: Ipilimumab followed by vemurafenib in a patient with brain metastasis. Internal Medicine. 2012;51(19):2819-23.
11. Barjaktarevic I, Qadir N, Suri A, Santamauro J, Stover D. Organizing pneumonia as a side-effect of ipilimumab treatment for malignant melanoma. Chest. 2012;1).
12. Barjaktarevic IZ, Qadir N, Suri A, Santamauro JT, Stover D. Organizing pneumonia as a side effect of ipilimumab treatment of melanoma. Chest. 2013;143(3):858-61.
13. Barnard ZR, Walcott BP, Kahle KT, Nahed BV, Coumans JV. Hyponatremia associated with Ipilimumab-induced hypophysitis. Med Oncol. 2012;29(1):374-7.
14. Bellutti M, Goppner D, Bonnekoh B, Franke I, Gollnick H. "SDRIFE"- flexural fixed Drug Eruption with toxic Immunotherapy with Ipilimumab in metastatic Melanoma. J Dtsch Dermatol Ges. 2013;11:154-5.
15. Berthod G, Lazor R, Letovanec I, Romano E, Noirez L, Mazza Stalder J, et al. Pulmonary sarcoid-like granulomatosis induced by ipilimumab. Journal of Clinical Oncology. 2012;30(17):e156-9.
16. Bhatia S, Huber BR, Upton MP, Thompson JA. Inflammatory enteric neuropathy with severe constipation after ipilimumab treatment for melanoma: a case report. Journal of Immunotherapy. 2009;32(2):203-5.
17. Bompaire F, Mateus C, Taillia H, De Greslan T, Lahutte M, Sallansonnet-Froment M, et al. Severe meningo-radiculo-neuritis associated with ipilimumab. Invest New Drugs. 2012;30(6):2407-10.

18. Borodic G, Hinkle DM, Cia Y. Drug-induced graves disease from CTLA-4 receptor suppression. Ophthal Plast Reconstr Surg. 2011;27(4):e87-8.
19. Borodic GE, Hinkle D. Ipilimumab-induced orbital inflammation resembling Graves disease with subsequent development of systemic hyperthyroidism from CTLA-4 receptor suppression. Ophthal Plast Reconstr Surg. 2014;30(1):83.
20. Borodic GE, Hinkle DM. Reply Re: "Drug-induced Graves disease from CTLA-4 receptor suppression". Ophthal Plast Reconstr Surg. 2013;29(3):241.
21. Bot I, Blank CU, Boogerd W, Brandsma D. Neurological immune-related adverse events of ipilimumab. Pract. 2013;13(4):278-80.
22. Bot I, Blank CU, Brandsma D. Clinical and radiological response of leptomeningeal melanoma after whole brain radiotherapy and ipilimumab. Journal of Neurology. 2012;259(9):1976-8.
23. Boyer A, Greillier L, Barazzutti H, Tomasini P, Barlesi F. [Ipilimumab and metastatic lung cancer: Can we change the natural history of the disease?]. Revue des maladies respiratoires. 2015.
24. Bryce J, Passoni C. Nursing management of patients with metastatic melanoma receiving ipilimumab. Oncol Nurs Forum. 2013;40(3):215-8.
25. Burke MM, Kluger HM, Golden M, Heller KN, Hoos A, Sznol M. Case Report: response to ipilimumab in a patient with HIV with metastatic melanoma. J Clin Oncol. 2011;29(32):e792-4.
26. Carlos G, Anforth R, Chou S, Clements A, Fernandez-Penas P. A case of bullous pemphigoid in a patient with metastatic melanoma treated with pembrolizumab. Melanoma research. 2015.
27. Carpenter KJ, Murtagh RD, Lilienfeld H, Weber J, Murtagh FR. Ipilimumab-induced hypophysitis: MR imaging findings. AJNR Am J Neuroradiol. 2009;30(9):1751-3.
28. Cavalcante L, Amin A, Lutzky J. Ipilimumab was safe and effective in two patients with metastatic melanoma and end-stage renal disease. Cancer Management and Research. 2015;7:47-50.
29. Cecchini M, Sznol M, Seropian S. Immune therapy of metastatic melanoma developing after allogeneic bone marrow transplant. Journal for ImmunoTherapy of Cancer. 2015;3(1).
30. Chan MMK, Kefford RF, Carlino M, Clements A, Manolios N. Arthritis and tenosynovitis associated with the anti-PD1 antibody pembrolizumab in metastatic melanoma. Journal of Immunotherapy. 2015;38(1):37-9.
31. Chargari C, Le Moulec S, Bonardel G, Foehrenbach H, Vedrine L. Ipilimumab in cancer patients: the issue of early metabolic response. Anticancer Drugs. 2013;24(3):324-6.
32. Chmiel KD, Suan D, Liddle C, Nankivell B, Ibrahim R, Bautista C, et al. Resolution of severe ipilimumab-induced hepatitis after antithymocyte globulin therapy. Journal of Clinical Oncology. 2011;29(9):e237-40.
33. Conry RM, Sullivan JC, Nabors LB. Ipilimumab-Induced Encephalopathy with a Reversible Splenial Lesion. Cancer Immunol Res. 2015;3(6):598-601.
34. Crews J, Agarwal A, Jack L, Xu D, Do DV, Nguyen QD. Ipilimumab-associated retinopathy. Ophthalmic Surgery Lasers and Imaging Retina. 2015;46(6):658-60.
35. Crosson JN, Laird PW, Debiec M, Bergstrom CS, Lawson DH, Yeh S. Vogt-koyanagi-harada-like syndrome after CTLA-4 inhibition with ipilimumab for metastatic melanoma. Journal of Immunotherapy. 2015;38(2):80-4.

36. De Felice K, Raffals L. High dose budesonide in steroid refractory ipilimumab-induced colitis. Inflammatory Bowel Diseases. 2013;19:S26.
37. De Hollanda A, Aranda GB, Mora M, Gaba L, Halperin I. Ipilimumab, a cause of autoimmune hypophysitis. [Spanish]. Endocrinologia y Nutricion. 2013;60(10):604-6.
38. De Sousa SM, Long GV, Tonks KT. Ipilimumab-induced hypophysitis: early Australian experience. Med J Aust. 2014;201(4):198-9.
39. Delyon J, Mateus C, Lambert T. Hemophilia A induced by ipilimumab. N Engl J Med. 2011;365(18):1747-8.
40. Di Giacomo AM, Danielli R, Guidoboni M, Calabro L, Carlucci D, Miracco C, et al. Therapeutic efficacy of ipilimumab, an anti-CTLA-4 monoclonal antibody, in patients with metastatic melanoma unresponsive to prior systemic treatments: clinical and immunological evidence from three patient cases. Cancer Immunol Immunother. 2009;58(8):1297-306.
41. Dick J, Enk A, Hassel JC. Long-lasting responses under treatment with ipilimumab: an argument against maintenance therapy? Dermatology. 2015;230(1):8-10.
42. Dillard T, Yedinak CG, Alumkal J, Fleseriu M. Anti-CTLA-4 antibody therapy associated autoimmune hypophysitis: serious immune related adverse events across a spectrum of cancer subtypes. Pituitary. 2010;13(1):29-38.
43. Dilling P, Walczak J, Pikiel P, Kruszewski WJ. Multiple colon perforation as a fatal complication during treatment of metastatic melanoma with ipilimumab - case report. Pol Przegl Chir. 2014;86(2):94-6.
44. Du Four S, Wilgenhof S, Duerinck J, Michotte A, Binst AV, Ridder MD, et al. Radiation necrosis of the brain in melanoma patients successfully treated with ipilimumab, three case studies. European Journal of Cancer. 2012;48(16):3045-51.
45. Du Rusquec P, Saint-Jean M, Brocard A, Peuvrel L, Khammari A, Quereux G, et al. Ipilimumab-induced autoimmune pancytopenia in a case of metastatic melanoma. Journal of Immunotherapy. 2014;37(6):348-50.
46. Eckert A, Schoeffler A, Dalle S, Phan A, Kiakouama L, Thomas L. Anti-CTLA4 monoclonal antibody induced sarcoidosis in a metastatic melanoma patient. Dermatology. 2009;218(1):69-70.
47. Eranki VG, Elhomsy G, Silverberg A, Albert S. Ipilimumab-associated hypophysitis-time course of MRI and hormonal changes. Endocrine Reviews. 2012;33 (3 MeetingAbstracts).
48. Eryilmaz MK, Mutlu H, Salim DK, Musri FY, Tural D, Bassorgun I, et al. Ipilimumab may increase the severity of cutenaous toxicity related to radiotherapy. Journal of oncology pharmacy practice : official publication of the International Society of Oncology Pharmacy Practitioners. 2015.
49. Fadel F, El Karoui K, Knebelmann B. Anti-CTLA4 antibody-induced lupus nephritis. N Engl J Med. 2009;361(2):211-2.
50. Fischli S, Allelein S, Zander T, Henzen C. [Endocrinologic side effects of oncologic treatment with anti-CTLA-4-antibodies]. Dtsch Med Wochenschr. 2014;139(19):996-1000.
51. Forde PM, Rock K, Wilson G, O'Byrne KJ. Ipilimumab-induced immune-related renal failure--a case report. Anticancer Research. 2012;32(10):4607-8.
52. Franzen D, Schad K, Dummer R, Russi EW. Severe acute respiratory distress syndrome due to ipilimumab. Eur Respir J. 2013;42(3):866-8.
53. Gao Y, Sharma S, Boparai R. Dire diarrhea: Ipilimumab induced colitis and its management. Journal of General Internal Medicine. 2014;29:S345.

54. Garcia-Varona A, Odze RD, Makrauer F. Lymphocytic colitis secondary to ipilimumab treatment. Inflammatory Bowel Diseases. 2013;19(2):E15-6.
55. Gaudy-Marqueste C, Monestier S, Franques J, Cantais E, Richard MA, Grob JJ. A severe case of ipilimumab-induced guillain-barre syndrome revealed by an occlusive enteric neuropathy: a differential diagnosis for ipilimumab-induced colitis. Journal of Immunotherapy. 2013;36(1):77-8.
56. Geisler BP, Raad RA, Esaian D, Sharon E, Schwartz DR. Apical ballooning and cardiomyopathy in a melanoma patient treated with ipilimumab: A case of takotsubo-like syndrome. Journal for ImmunoTherapy of Cancer. 2015;3(1).
57. Gentile NM, D'Souza A, Fujii LL, Wu TT, Murray JA. Association between ipilimumab and celiac disease. Mayo Clin Proc. 2013;88(4):414-7.
58. Gerdes LA, Junker A, Berking C, Tietze J, Straube A, Kreth FW, et al. Multiple sclerosis as immune related adverse event after ipilimumab treatment in metastatic melanoma. Multiple Sclerosis. 2014;1):454.
59. Gil SM, Aparicio M, Bertini K, Rodriguez F, Sankowicz S, Ballarino C. Autoimmune hypophysitis due to ipilimumab. Endocrine Reviews. 2013;1).
60. Gilardi L, Colandrea M, Vassallo S, Travaini LL, Paganelli G. Ipilimumab-induced immunomediated adverse events: possible pitfalls in (18)F-FDG PET/CT interpretation. Clin Nucl Med. 2014;39(5):472-4.
61. Goethals L, Wilgenhof S, De Geeter F, Everaert H, Neyns B. 18F-FDG PET/CT imaging of an anti-CTLA-4 antibody-associated autoimmune pancolitis. European Journal of Nuclear Medicine and Molecular Imaging. 2011;38(7):1390-1.
62. Goldstein BL, Gedmintas L, Todd DJ. Drug-associated polymyalgia rheumatica/giant cell arteritis occurring in two patients after treatment with ipilimumab, an antagonist of ctla-4. Arthritis rheumatol. 2014;66(3):768-9.
63. Goldstein BL, Gedmintas L, Todd DJ. Concise communication. Arthritis and Rheumatology. 2014;66(3):768-9.
64. Gordon IO, Wade T, Chin K, Dickstein J, Gajewski TF. Immune-mediated red cell aplasia after anti-CTLA-4 immunotherapy for metastatic melanoma. Cancer Immunol Immunother. 2009;58(8):1351-3.
65. Gorka E, Balatoni T, Porneczy E, Czirbesz K, Bozoky F, Panczel G, et al. Immune-related, extremely rare adverse effect of ipilimumab: Serious autoimmune orbital inflammation after ipilimumab reinduction: A case report. Pigment Cell and Melanoma Research. 2014;27 (6):1194.
66. Gormley R, Wanat K, Elenitsas R, Giles J, McGettigan S, Schuchter L, et al. Ipilimumab-associated Sweet syndrome in a melanoma patient. J Am Acad Dermatol. 2014;71(5):e211-3.
67. Haider A, O'Riordan K. Ipilimumab-induced colonic perforation. American Journal of Gastroenterology. 2014;109:S421-S2.
68. Hanrahan P, Van Der Westhuizen A, Collins S, Owens D, Hersey P. Delayed autoimmune effects in patients treated with antibodies against the checkpoint inhibitor PD1. JDDG - Journal of the German Society of Dermatology. 2013;11:16.
69. Harmankaya K, Erasim C, Koelblinger C, Ibrahim R, Hoos A, Pehamberger H, et al. Continuous systemic corticosteroids do not affect the ongoing regression of metastatic melanoma for more than two years following ipilimumab therapy. Med Oncol. 2011;28(4):1140-4.
70. Henderson AD, Thomas DA. A Case Report of Orbital Inflammatory Syndrome Secondary to Ipilimumab. Ophthalmic plastic and reconstructive surgery. 2014.

71. Herlopian A, Mahmoud F, David R, Samant R, Gokden M, Hutchins L, et al. Neurological complications of ipilimumab therapy in patients with metastatic melanoma. Neurology. 2015;84.
72. Hinds AM, Ahmad DS, Muenster JE, Berg ZM, Lopez KT, Holly JSL, et al. Ipilimumab-induced colitis: A rare but serious side effect. Endoscopy. 2014;46(SUPPL 1):E308-E9.
73. Hrgovic I, Winkelmann R, Vogl TJ, Von Wagner M, Youzouri HE, Kaufmann R, et al. Sigmoid diverticulosis - a risk factor for perforation of the sigmoid colon due to ipilimumab-associated enterocolitis? JDDG - Journal of the German Society of Dermatology. 2015;13(6):575-7.
74. Hundorfean G, Atreya R, Agaimy A, Heinzerling L, Kampgen E, Schuler G, et al. Fluorescein-guided confocal laser endomicroscopy for the detection of ipilimumab-induced colitis. Endoscopy. 2012;44 Suppl 2 UCTN:E78-9.
75. Hunter G, Voll C, Robinson CA. Autoimmune inflammatory myopathy after treatment with ipilimumab. Can J Neurol Sci. 2009;36(4):518-20.
76. Izzedine H, Gueutin V, Gharbi C, Mateus C, Robert C, Routier E, et al. Kidney injuries related to ipilimumab. Invest New Drugs. 2014;32(4):769-73.
77. Jinnur P, Lim KG. Severe Acute Orthopnea: Ipilimumab-Induced Bilateral Phrenic Nerve Neuropathy. Lung. 2015;193(4):611-3.
78. Johnson DB, Wallender EK, Cohen DN, Likhari SS, Zwerner JP, Powers JG, et al. Severe cutaneous and neurologic toxicity in melanoma patients during vemurafenib administration following anti-PD-1 therapy. Cancer Immunol Res. 2013;1(6):373-7.
79. Joseph RW, Cappel M, Goedjen B, Gordon M, Kirsch B, Gilstrap C, et al. Lichenoid Dermatitis in Three Patients with Metastatic Melanoma Treated with Anti-PD-1 Therapy. Cancer Immunol Res. 2015;3(1):18-22.
80. Juszczak A, Gupta A, Karavitaki N, Middleton MR, Grossman AB. Ipilimumab: a novel immunomodulating therapy causing autoimmune hypophysitis: a case report and review. Eur. 2012;167(1):1-5.
81. Kaehler KC, Egberts F, Lorigan P, Hauschild A. Anti-CTLA-4 therapy-related autoimmune hypophysitis in a melanoma patient. Melanoma Research. 2009;19(5):333-4.
82. Kamil F, Cohen M, Kumta N, Wan D. Ipilimumab-induced colitis in a patient with ulcerative colitis and lung cancer. American Journal of Gastroenterology. 2013;108:S404.
83. Khirfan K, Kistin M. Colitis associated with biological agents. Dig Dis Sci. 2014;59(6):1112-4.
84. Kleiner DE, Berman D. Pathologic changes in ipilimumab-related hepatitis in patients with metastatic melanoma. Dig Dis Sci. 2012;57(8):2233-40.
85. Koch C, Paetzold S, Trojan J. Enterocolitis in a patient being treated with ipilimumab for metastatic melanoma. Gastroenterology. 2012;143(2):298, 504, 5.
86. Kopecky J, Trojanova P, Kubecek O, Kopecky O. Treatment possibilities of ipilimumab-induced thrombocytopenia--case study and literature review. Jpn J Clin Oncol. 2015;45(4):381-4.
87. Kwun S, Lukacova-Zib I, Gopalakrishnan G. Central adrenal insufficiency and hypothyroidism after ipilimumab treatment. Endocrine Reviews. 2012;33 (3 MeetingAbstracts).
88. Kyllo RL, Parker MK, Rosman I, Musiek AC. Ipilimumab-associated Sweet syndrome in a patient with high-risk melanoma. J Am Acad Dermatol. 2014;70(4):e85-6.
89. Lambert Smith F, Wisell J, Brown M. Advanced acral melanoma. JAAD Case Reports. 2015;1(3):166-8.

90. Lammert A, Schneider HJ, Bergmann T, Benck U, Kramer BK, Gartner R, et al. Hypophysitis caused by ipilimumab in cancer patients: hormone replacement or immunosuppressive therapy. Exp Clin Endocrinol Diabetes. 2013;121(10):581-7.
91. Lecouflet M, Verschoore M, Giard C, Gohier P, Le Corre Y, Milea D, et al. Orbital myositis associated with ipilimumab. Melanoma Research. 2011;21:e24.
92. Lecouflet M, Verschoore M, Giard C, Gohier P, Le Corre Y, Milea D, et al. [Orbital myositis associated with ipilimumab]. Ann Dermatol Venereol. 2013;140(6-7):448-51.
93. Ledezma B, Binder S, Hamid O. Atypical clinical response patterns to ipilimumab. Clin J Oncol Nurs. 2011;15(4):393-403.
94. Leonard D, Desai U. Hyponatremia and hypopituitarism secondary to Ipilimumab. American Journal of Kidney Diseases. 2013;61 (4):A59.
95. Liao B, Shroff S, Kamiya-Matsuoka C, Tummala S. Atypical neurological complications of ipilimumab therapy in patients with metastatic melanoma. Neuro-Oncology. 2014;16(4):589-93.
96. Liao B, Shroff S, Kamiya-Matsuoka C, Tummala S. Atypical neurological complications of ipilimumab therapy in patients with a metastatic melanoma. Neurology. 2014;1).
97. Lim JL, Dahiya M, Burgin S. Intertriginous and follicular eruption to anticytotoxic T-lymphocyte antigen 4 monoclonal antibody. J Am Acad Dermatol. 2008;59(2 Suppl 1):S60-1.
98. Lipson EJ, Bodell MA, Kraus ES, Sharfman WH. Successful administration of ipilimumab to two kidney transplantation patients with metastatic melanoma. Journal of Clinical Oncology. 2014;32(19):e69-e71.
99. Loochtan AI, Nickolich MS, Hobson-Webb LD. Myasthenia gravis associated with ipilimumab and nivolumab in the treatment of small cell lung cancer. Muscle and Nerve. 2015;52(2):307-8.
100. Ludlow SP, Kay N. Delayed Dermatologic Hypersensitivity Reaction Secondary to Ipilimumab. Journal of immunotherapy (Hagerstown, Md : 1997). 2015.
101. Luke JJ, Lezcano C, Hodi FS, Murphy GF. Antitumor granuloma formation by CD4+ T cells in a patient with rapidly progressive melanoma experiencing spiking fevers, neuropathy, and other immune-related toxicity after treatment with ipilimumab. Journal of Clinical Oncology. 2015;33(6):e32-5.
102. Lyall A, Vargas HA, Carvajal RD, Ulaner G. Ipilimumab-induced colitis on FDG PET/CT. Clin Nucl Med. 2012;37(6):629-30.
103. Mahzari M, Liu D, Arnaout A, Lochnan H. Immune checkpoint inhibitor therapy associated hypophysitis. Clin Med Insights Endocrinol Diabetes. 2015;8:21-8.
104. Mailleux M, Cornelis F, Colin G, Baurain JF. Unusual pulmonary toxicity of ipilimumab treated by macrolides. Acta clinica Belgica. 2015:2295333715y0000000047.
105. Majchel D, Korytkowski MT. Anticytotoxic T-lymphocyte antigen-4 induced autoimmune hypophysitis: a case report and literature review. case report. 2015;2015:570293.
106. Manousakis G, Koch J, Sommerville RB, El-Dokla A, Harms MB, Al-Lozi MT, et al. Multifocal radiculoneuropathy during ipilimumab treatment of melanoma. Muscle Nerve. 2013;48(3):440-4.
107. Manusow JS, Khoja L, Pesin N, Joshua AM, Mandelcorn ED. Retinal vasculitis and ocular vitreous metastasis following complete response to PD-1 inhibition in a patient with metastatic cutaneous melanoma. Journal for ImmunoTherapy of Cancer. 2015;2(1).

108. Marlier J, Cocquyt V, Brochez L, Van Belle S, Kruse V. Ipilimumab, not just another anti-cancer therapy: hypophysitis as side effect illustrated by four case-reports. Endocrine. 2014;47(3):878-83.
109. Martinez-Balzano C. Management of ipilimumab-induced colitis with concurrent clostridium difficile infection. American Journal of Gastroenterology. 2012;107:S468.
110. Martin-Liberal J, Furness AJ, Joshi K, Peggs KS, Quezada SA, Larkin J. Anti-programmed cell death-1 therapy and insulin-dependent diabetes: a case report. Cancer immunology, immunotherapy : CII. 2015.
111. Maur M, Tomasello C, Frassoldati A, Dieci MV, Barbieri E, Conte P. Posterior reversible encephalopathy syndrome during ipilimumab therapy for malignant melanoma. Journal of Clinical Oncology. 2012;30(6):e76-8.
112. McElnea E, Ni Mhealoid A, Moran S, Kelly R, Fulcher T. Thyroid-like ophthalmopathy in a euthyroid patient receiving Ipilimumab. Orbit. 2014;33(6):424-7.
113. Merrill SP, Reynolds P, Kalra A, Biehl J, Vandivier RW, Mueller SW. Early administration of infliximab for severe ipilimumab-related diarrhea in a critically ill patient. Ann Pharmacother. 2014;48(6):806-10.
114. Min L, Hodi FS. Anti-PD1 following ipilimumab for mucosal melanoma: durable tumor response associated with severe hypothyroidism and rhabdomyolysis. Cancer Immunol Res. 2014;2(1):15-8.
115. Min L, Ibrahim N. Ipilimumab-induced autoimmune adrenalitis. Lancet Diabetes Endocrinol. 2013;1(3):e15.
116. Min L, Vaidya A, Becker C. Thyroid autoimmunity and ophthalmopathy related to melanoma biological therapy. Eur. 2011;164(2):303-7.
117. Minor DR, Puzanov I, Callahan MK, Hug BA, Hoos A. Severe gastrointestinal toxicity with administration of trametinib in combination with dabrafenib and ipilimumab. Pigment cell & melanoma research. 2015.
118. Minor DR, Bunker SR, Doyle J. Lymphocytic vasculitis of the uterus in a patient with melanoma receiving ipilimumab. Journal of Clinical Oncology. 2013;31(20):e356.
119. Minor DR, Chin K, Kashani-Sabet M. Infliximab in the treatment of anti-CTLA4 antibody (ipilimumab) induced immune-related colitis. Cancer Biother Radiopharm. 2009;24(3):321-5.
120. Mis L, Clarke JM. Ipilimumab-induced pneumonitis: A case report. Journal of Pharmacy Technology. 2013;29(2):94-8.
121. Miserocchi E, Cimminiello C, Mazzola M, Russo V, Modorati GM. New-onset uveitis during CTLA-4 blockade therapy with ipilimumab in metastatic melanoma patient. Canadian Journal of Ophthalmology. 2015;50(1):e2-e4.
122. Mitchell KA, Kluger H, Sznol M, Hartman DJ. Ipilimumab-induced perforating colitis. J Clin Gastroenterol. 2013;47(9):781-5.
123. Modjtahedi BS, Maibach H, Park S. Multifocal bilateral choroidal neovascularization in a patient on ipilimumab for metastatic melanoma. Cutan. 2013;32(4):341-3.
124. Morales RE, Shoushtari AN, Walsh MM, Grewal P, Lipson EJ, Carvajal RD. Safety and efficacy of ipilimumab to treat advanced melanoma in the setting of liver transplantation. Journal for Immunotherapy of Cancer. 2015;3:22.
125. Muhammad A, Reed J, Vidyarthi G, Boyd W. Drug induced colon injury (DICI): A case of ipilimumab induced immune related colitis. American Journal of Gastroenterology. 2012;107:S456.

126. Munoz J, Guillot B, Girard C, Dereure O, Du-Thanh A. First report of ipilimumab-induced Grover disease. British Journal of Dermatology. 2014;171(5):1236-7.
127. Murakami T, Pugh J, Ozden N, Trowers E. Mimicking inflammatory bowel disease during treatment of metastatic melanoma: Immune mediated ipilimumab-induced colitis. American Journal of Gastroenterology. 2013;108:S386-S7.
128. Murer C, Goldinger SM, Marques Maggio E, Bjasch D, Stieger P, Mihic-Probst D, et al. Hyponatraemia and tachyarrhythmia during ipilimumab: Lessons from the autopsy. JDDG - Journal of the German Society of Dermatology. 2013;11:77.
129. Murphy KP, Kennedy MP, Barry JE, O'Regan KN, Power DG. New-onset mediastinal and central nervous system sarcoidosis in a patient with metastatic melanoma undergoing CTLA4 monoclonal antibody treatment. Oncology Research and Treatment. 2014;37(6):351-3.
130. Nallapaneni NN, Mourya R, Bhatt VR, Malhotra S, Ganti AK, Tendulkar KK. Ipilimumab-induced hypophysitis and uveitis in a patient with metastatic melanoma and a history of ipilimumab-induced skin rash. J. 2014;12(8):1077-81.
131. Nancey S, Boschetti G, Cotte E, Ruel K, Almeras T, Chauvenet M, et al. Blockade of cytotoxic T-lymphocyte antigen-4 by ipilimumab is associated with a profound long-lasting depletion of Foxp3+ regulatory T cells: a mechanistic explanation for ipilimumab-induced severe enterocolitis? Inflammatory Bowel Diseases. 2012;18(8):E1598-600.
132. Narita T, Oiso N, Taketomo Y, Okahashi K, Yamauchi K, Sato M, et al. Serological aggravation of autoimmune thyroid disease in two cases receiving nivolumab. The Journal of dermatology. 2015.
133. Nishino M, Sholl LM, Hodi FS, Hatabu H, Ramaiya NH. Anti-PD-1-Related Pneumonitis during Cancer Immunotherapy. N Engl J Med. 2015;373(3):288-90.
134. Noirez L, Berthod G, Letovanec I, Mazza Stalder J, Michielin O, Lazor R. Pulmonary granulomatosis and organising pneumonia induced by ipilimumab. Respiration. 2011;82 (1):97-8.
135. Ohtsuka M, Miura T, Mori T, Ishikawa M, Yamamoto T. Occurrence of psoriasiform eruption during nivolumab therapy for primary oral mucosal melanoma. JAMA Dermatol. 2015;151(7):797-9.
136. O'Kane GM, Lyons TG, Colleran GC, Ahmad MW, Alken S, Kavanagh EC, et al. Late-onset paraplegia after complete response to two cycles of ipilimumab for metastatic melanoma. Oncology Research and Treatment. 2014;37(12):757-60.
137. Pages C, Gornet JM, Monsel G, Allez M, Bertheau P, Bagot M, et al. Ipilimumab-induced acute severe colitis treated by infliximab. Melanoma Research. 2013;23(3):227-30.
138. Pedersen M, Andersen R, Norgaard P, Jacobsen S, Thielsen P, thor Straten P, et al. Successful treatment with Ipilimumab and Interleukin-2 in two patients with metastatic melanoma and systemic autoimmune disease. Cancer Immunol Immunother. 2014;63(12):1341-6.
139. Penumetsa K, Ponugoti S, Reynolds G. Ipilimumab-induced colitis with concomitant clostridium difficile infection. American Journal of Gastroenterology. 2013;108:S392.
140. Pintova S, Sidhu H, Friedlander PA, Holcombe RF. Sweet's syndrome in a patient with metastatic melanoma after ipilimumab therapy. Melanoma Research. 2013;23(6):498-501.
141. Plachouri KM, Mohr M, Sunderkotter C, Weishaupt C. Induction of muscular sarcoidosis in a metastastic melanoma patient treated with ipilimumab. JDDG - Journal of the German Society of Dermatology. 2012;10 (11):861.

142. Weishaupt C, Mohr M, Sunderkotter C. Muscular sarcoidosis in a metastastic melanoma patient treated with ipilimumab. JDDG - Journal of the German Society of Dermatology. 2012;10 (9):683.
143. Pocha C, Roat J, Viskocil K. Immune-mediated colitis: important to recognize and treat. J Crohns Colitis. 2014;8(2):181-2.
144. Postow MA, Callahan MK, Barker CA, Yamada Y, Yuan J, Kitano S, et al. Immunologic correlates of the abscopal effect in a patient with melanoma. N Engl J Med. 2012;366(10):925-31.
145. Rastogi P, Sultan M, Charabaty AJ, Atkins MB, Mattar MC. Ipilimumab associated colitis: An IpiColitis case series at MedStar Georgetown University Hospital. World Journal of Gastroenterology. 2015;21(14):4373-8.
146. Ratuapli SK, Depetris G, Shepard B, Nguyen CC. Severe colitis caused by biologics: what an endoscopist needs to know. Minerva Gastroenterol Dietol. 2014;60(4):287-90.
147. Reule RB, North JP. Cutaneous and pulmonary sarcoidosis-like reaction associated with ipilimumab. J Am Acad Dermatol. 2013;69(5):e272-3.
148. Robinson MR, Chan CC, Yang JC, Rubin BI, Gracia GJ, Sen HN, et al. Cytotoxic T lymphocyte-associated antigen 4 blockade in patients with metastatic melanoma: A new cause of uveitis. Journal of Immunotherapy. 2004;27(6):478-9.
149. Rodrigues BT, Otty Z, Sangla K, Shenoy VV. Ipilimumab-induced autoimmune hypophysitis: a differential for sellar mass lesions. Endocrinol Diabetes Metab Case Rep. 2014;2014:140098.
150. Rudolph B, Groffik A, Muller-Brenne T, Von Stebut E, Grabbe S, Loquai C. Severe colitis with pyoderma gangrenosum after ipilimumab treatment in a melanoma patient with colostomy-a therapeutic challenge. JDDG - Journal of the German Society of Dermatology. 2011;9 (9):788.
151. Rudolph BM, Staib F, Von Stebut E, Hainz M, Grabbe S, Loquai C. Neutrophilic disease of the skin and intestines after ipilimumab treatment for malignant melanoma - simultaneous occurrence of pyoderma gangrenosum and colitis. European journal of dermatology : EJD. 2014;24(2):268-9.
152. Saenger YM, Wolchok JD. The heterogeneity of the kinetics of response to ipilimumab in metastatic melanoma: Patient cases. Cancer Immun. 2008;8(17).
153. Sampath AM, Khorasani-Zadeh A, Hajar N, Rawlins S. A rare presentation of severe colitis with ipilimumab therapy. American Journal of Gastroenterology. 2013;108:S399.
154. Schartz NEC, Farges C, Madelaine I, Bruzzoni H, Calvo F, Hoos A, et al. Complete regression of a previously untreated melanoma brain metastasis with ipilimumab. Melanoma Research. 2010;20(3):247-50.
155. Schleder S, Schreml S, Heiss P. [Ipilimumab-induced hypophysitis]. ROFO Fortschr Geb Rontgenstr Nuklearmed. 2013;185(3):268-9.
156. Shah R, Kapoor E, Glenna M, Markovic S. Paradoxical breathing: An important physical exam finding in a patient with metastatic melanoma. Journal of General Internal Medicine. 2014;29:S405.
157. Sheik Ali S, Goddard AL, Luke JJ, Donahue H, Todd DJ, Werchniak A, et al. Drug-associated dermatomyositis following ipilimumab therapy: a novel immune-mediated adverse event associated with cytotoxic T-lymphocyte antigen 4 blockade. JAMA Dermatol. 2015;151(2):195-9.

158. Shivaprasad S, Tummala S. Chronic inflammatory polyradiculonneuropathy (CIDP) secondary to ipilimumab therapy in a patient with metastatic melanoma. Journal of Clinical Neuromuscular Disease. 2013;14 (3):146.
159. Slingerland M, Nortier JW, Veenendaal RA, Kapiteijn E. Severe colitis while responding to ipilimumab in metastatic melanoma. Acta Oncol. 2012;51(6):805-7.
160. Sohrab MA, Desai RU, Chambers CB, Lissner GS. Re: "Drug-induced Graves disease from CTLA-4 receptor suppression". Ophthal Plast Reconstr Surg. 2013;29(3):239-40.
161. Sprung B, Sathyamurthy A, Lewis J, Cellini C, DeCross A, Kaul V, et al. A case of perforating ipilimumab-induced autoimmune colitis. American Journal of Gastroenterology. 2014;109:S409.
162. Sun J, Schiffman J, Raghunath A, Ng Tang D, Chen H, Sharma P. Concurrent decrease in IL-10 with development of immune-related adverse events in a patient treated with anti-CTLA-4 therapy. Cancer Immun. 2008;8:9.
163. Thaipisuttikul I, Avila E. Peripheral neuropathy associated with ipilimumab: A report of two cases. Neuro-Oncology. 2011;13:iii39.
164. Thaipisuttikul I, Chapman P, Avila EK. Peripheral neuropathy associated with ipilimumab: A report of 2 cases. Journal of Immunotherapy. 2015;38(2):77-9.
165. Thajudeen B, Koppula S, Madhrira M. Ipilimumab induced granulomatous interstitial nephritis. American Journal of Kidney Diseases. 2012;59 (4):A85.
166. Thajudeen B, Madhrira M, Bracamonte E, Cranmer LD. Ipilimumab granulomatous interstitial nephritis. American Journal of Therapeutics. 2015;22(3):e84-e7.
167. Thomsen HH. [Lymphocytic hypophysitis due to ipilimumap therapy]. Ugeskr Laeger. 2012;174(26):1829-30.
168. Tissot C, Carsin A, Freymond N, Pacheco Y, Devouassoux G. Sarcoidosis complicating anti-cytotoxic T-lymphocyte-associated antigen-4 monoclonal antibody biotherapy. Eur Respir J. 2013;41(1):246-7.
169. Tiu C, Pezaro C, Davis ID, Grossmann M, Parente P. Early recognition of ipilimumab-related autoimmune hypophysitis in patients with metastatic melanoma: Case studies and recommendations for management. Asia-Pacific Journal of Clinical Oncology. 2015;11(2):190-4.
170. Toumeh A, Sakhi R, Shah S, Arudra SK, De Las Casas LE, Skeel RT. Ipilimumab-Induced Granulomatous Disease Occurring Simultaneously With Disease Progression in a Patient With Metastatic Melanoma. American journal of therapeutics. 2015.
171. Tsaknakis B, Schaefer IM, Schworer H, Sahlmann CO, Thoms KM, Blaschke M, et al. Long-lasting complete response of metastatic melanoma to ipilimumab with analysis of the resident immune cells. Med Oncol. 2014;31(1).
172. Tsiaras A. Case of the month. Autoimmune colitis secondary to CTLA-4 blockade. Jaapa. 2011;24(8):68.
173. Uslu U, Agaimy A, Hundorfean G, Harrer T, Schuler G, Heinzerling L. Autoimmune colitis and subsequent CMV-induced hepatitis after treatment with ipilimumab. Journal of Immunotherapy. 2015;38(5):212-5.
174. van der Hiel B, Blank CU, Haanen JB, Stokkel MP. Detection of early onset of hypophysitis by (18)F-FDG PET-CT in a patient with advanced stage melanoma treated with ipilimumab. Clin Nucl Med. 2013;38(4):e182-4.

175. Van Rooij N, Van Buuren MM, Philips D, Velds A, Toebes M, Heemskerk B, et al. Tumor exome analysis reveals neoantigen-specific T-cell reactivity in an ipilimumab-responsive melanoma. Journal of Clinical Oncology. 2013;31(32):e439-e42.
176. Venditti O, De Lisi D, Caricato M, Caputo D, Capolupo GT, Taffon C, et al. Ipilimumab and immune-mediated adverse events: a case report of anti-CTLA4 induced ileitis. BMC Cancer. 2015;15:5.
177. Victoria Martinez AM, Estela Cubells JR, Cubells Sanchez L, Oliver Martinez V, Alegre De Miguel V. Ipilimumab-induced poliosis. [Spanish]. Medicina Clinica. 2014;142(5):234.
178. Vogel WV, Guislain A, Kvistborg P, Schumacher TN, Haanen JB, Blank CU. Ipilimumab-induced sarcoidosis in a patient with metastatic melanoma undergoing complete remission. Journal of Clinical Oncology. 2012;30(2):e7-e10.
179. Voskens C, Cavallaro A, Erdmann M, Dippel O, Kaempgen E, Schuler G, et al. Anti-cytotoxic T-cell lymphocyte antigen-4-induced regression of spinal cord metastases in association with renal failure, atypical pneumonia, vision loss, and hearing loss. Journal of Clinical Oncology. 2012;30(33):e356-e7.
180. Wilgenhof S, Four SD, Everaert H, Neyns B. Patterns of response in patients with pretreated metastatic melanoma who received ipilimumab 3 mg/kg in a European expanded access program: Five illustrative case reports. Cancer Investigation. 2012;30(10):712-20.
181. Wilgenhof S, Morlion V, Seghers AC, Du Four S, Vanderlinden E, Hanon S, et al. Sarcoidosis in a patient with metastatic melanoma sequentially treated with anti-CTLA-4 monoclonal antibody and selective BRAF inhibitor. Anticancer Research. 2012;32(4):1355-9.
182. Wilgenhof S, Neyns B. Anti-CTLA-4 antibody-induced Guillain-Barre syndrome in a melanoma patient. Ann Oncol. 2011;22(4):991-3.
183. Wilgenhof S, Pierret L, Corthals J, Van Nuffel AMT, Heirman C, Roelandt T, et al. Restoration of tumor equilibrium after immunotherapy for advanced melanoma: Three illustrative cases. Melanoma Research. 2011;21(2):152-9.
184. Wozniak S, Mackiewicz-Wysocka M, Krokowicz L, Kwinta L, Mackiewicz J. Febrile neutropenia in a metastatic melanoma patient treated with ipilimumab - case report. Oncology Research and Treatment. 2015;38(3):105-8.
185. Wyluda EJ, Cheng J, Schell TD, Haley JS, Mallon C, Neves RI, et al. Durable complete responses off all treatment in patients with metastatic malignant melanoma after sequential immunotherapy followed by a finite course of BRAF inhibitor therapy. Cancer biology & therapy. 2015:0.
186. Yarze J, Stoutenburg J. Ipilimumab-associated colitis. American Journal of Gastroenterology. 2013;108:S357.
187. Yeh OL, Francis CE. Ipilimumab-associated bilateral optic neuropathy. Journal of Neuro-Ophthalmology. 2015;35(2):144-7.
188. Yu C, Chopra IJ, Ha E. A novel melanoma therapy stirs up a storm: ipilimumab-induced thyrotoxicosis. Endocrinol Diabetes Metab Case Rep. 2015;2015:140092.
189. Yuan J, Page DB, Ku GY, Li Y, Mu Z, Ariyan C, et al. Correlation of clinical and immunological data in a metastatic melanoma patient with heterogeneous tumor responses to ipilimumab therapy. Cancer Immun. 2010;10(07).
190. Yun S, Vincelette ND, Mansour I, Hariri D, Motamed S. Late onset ipilimumab-induced pericarditis and pericardial effusion: A rare but life threatening complication. Case Reports in Oncological Medicine. 2015;2015(794842).

191. Zmeili O, Samantray J. Anti-CTLA-4 antibody therapy causing autoimmune hypophysitis in a patient with a longstanding history of hashimotos's thyroiditis. Endocrine Reviews. 2013;1).
